# Exploration of the causal effects of leukocyte telomere length and four gastrointestinal diseases: a two-sample bidirectional Mendelian randomization study

**DOI:** 10.1186/s12876-023-03081-y

**Published:** 2023-12-18

**Authors:** Haikuo Wang, Xiaolin Chen, Siming Wang, Heyun Zhang

**Affiliations:** 1 Department of Hepatobiliary Surgery, Sun Yat-sen Memorial Hospital, Guangzhou, Guangdong China; 2 Breast Tumor Center, Sun Yat-sen Memorial Hospital, Guangzhou, Guangdong China

**Keywords:** Mendelian randomization, Single nucleotide polymorphism, Leukocyte telomere length, Gastrointestinal diseases

## Abstract

**Background:**

To explore the underlying causality between leukocyte telomere length (LTL) and four gastrointestinal diseases, we designed a two-sample bidirectional Mendelian randomization study.

**Methods:**

Two-sample Mendelian randomization (MR) was used to explore genetic causality between LTL and four gastrointestinal diseases, including irritable bowel syndrome (IBS), gastroesophageal reflux disease (GERD), gastrointestinal ulcers disease (GUD), and nonalcoholic fatty liver disease (NAFLD). We utilized inverse-variance weighted (IVW) as the primary method for MR analysis. Supplementary analyses were conducted using methods such as MR-Egger regression, weighted-median, Maximum Likelihood (MaxLik), Robust adjusted profile score (MR-RAPS), Contamination mixture (ConMix), and MR-mix. Cochran’s Q was calculated to check for heterogeneity. The MR-Egger regression and MR pleiotropy residual sum and outlier (MR-PRESSO) were detected for pleiotropy.

**Results:**

The IVW analysis suggests that there may be a potential causal relationship between LTL and two diseases (odds ratio (OR): 1.062; 95% confidence interval (CI): 1.003, 1.124; *p* = 0.038 for IBS and OR: 0.889; 95% CI: 0.798, 0.990; *p* = 0.032 for GERD). However, other methods do not entirely align with the results of the IVW analysis. In the reverse MR analysis, we did not find statistically significant associations between LTL and these four diseases.

**Conclusion:**

The current evidence does not definitively rule out a causal relationship between LTL and these four gastrointestinal diseases but suggests a potential association between LTL and IBS, or LTL and GERD. Exploring the relationship between gastrointestinal diseases and LTL may offer new insights into the onset, progression, and treatment of these diseases.

**Supplementary Information:**

The online version contains supplementary material available at 10.1186/s12876-023-03081-y.

## Background

The escalating burden on healthcare services associated with non-neoplastic gastrointestinal diseases, such as irritable bowel syndrome (IBS), gastroesophageal reflux disease (GERD), peptic ulcer disease (PUD), and non-alcoholic fatty liver disease (NAFLD), has become increasingly pronounced. These conditions exhibit a high prevalence within the population, resulting in significant distress in daily life and potentially leading to more severe complications. Nevertheless, their etiology and pathogenesis remain incompletely understood. Mendelian Randomization (MR) researchs have indicated an association between leukocyte telomere length (LTL) and the onset or progression of diverse diseases, both neoplastic and non-neoplastic which provides a new perspective for understanding the underlying causes of diseases through genetic factors [1–3].

Telomeres are complex of repetitive sequences and proteins located at the ends of chromosomes, consisting of a repeating DNA base sequence “TTAGGG” and telomeric proteins [4]. Telomere length varies among different tissues within the same individual, and the telomere length of white blood cells in the blood exhibits a strong correlation with the telomere length of other tissues. Therefore, LTL can serve as a proxy for the telomere length of other tissues [5]. Leukocyte telomere is one of the important indicators to measure cell aging and lifespan, and the change of its length is related to disease occurrence and psychosocial factors [6, 7]. Zhang et al. discovered a significant reduction in LTL in patients with IBS who also had depression. Interestingly, patients with a longer duration of antidepressant treatment exhibited longer telomeres [8]. Souza et al. observed that, in two groups with no statistically significant differences in age and telomerase activity levels, the telomeres in the distal esophageal squamous epithelium of patients with GERD were significantly shorter than those in patients without GERD [9].Conflicting findings exist regarding the relationship between telomere length and the occurrence of non-alcoholic fatty liver disease (NAFLD). Previous study had shown that telomere length of hepatic cells and peripheral leukocytes is shorter in patients with NAFLD [10, 11]. However, in a comprehensive population study in the United States, no correlation was found between telomere length and NAFLD. This discrepancy may be attributed to the impact of confounding factors such as age and ethnicity [12]. The relationship between LTL and peptic ulcer disease has not been reported.

Due to the susceptibility of observational studies exploring causality to confounding factors and inverse causal relationships, it is challenging to definitively establish whether changes in leukocyte telomere length are causally related to the occurrence of these four gastrointestinal disorders using observational studies. Based on Mendel’s second law of inheritance, parental alleles are randomly assigned to offspring at conception, and specific genetic mutations follow this guideline. The disclosure of genome-wide association study statistics (GWAS) data enables the grouping of potentially exposed populations using randomly assigned single nucleotide polymorphisms (SNPs), a process reminiscent of randomized controlled studies. This approach effectively mitigates the impact of confounding factors, diminishes the occurrence of bias to a certain extent, and yields more reliable results [13, 14].

In this study, we employed a two-sample bidirectional Mendelian randomization analysis to explore the causal relationship between LTL and four chronic digestive diseases from a genetic perspective. We anticipate that our findings will offer new insights into the potential mechanisms and treatment approaches for these diseases.

## Methods

### Study design

**Fig. 1:**
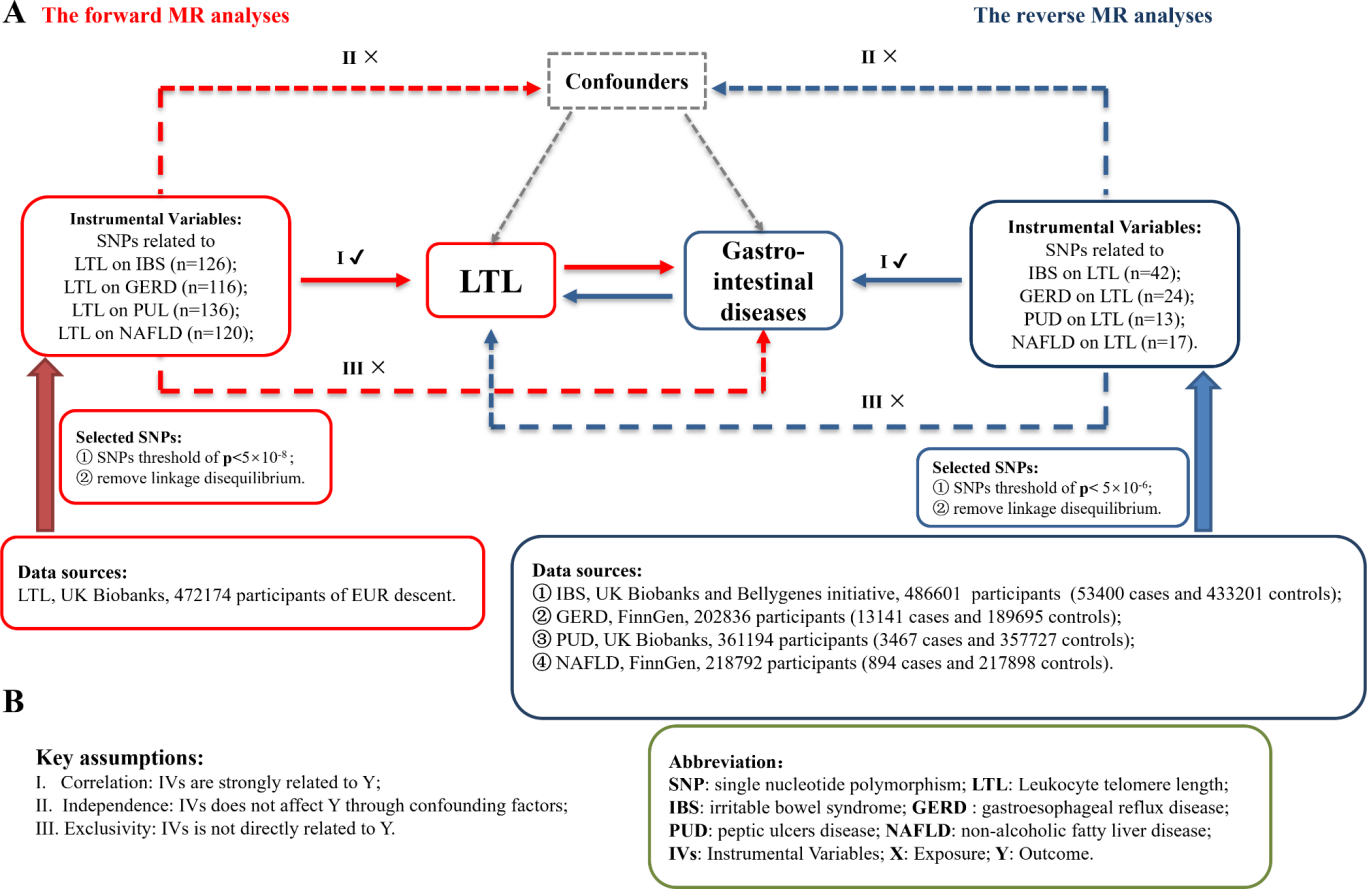
Mendelian Randomization Flowchart. Panel: (**A**) The red lines and rectangles illustrate the forward analysis with LTL as the exposure and chronic gastrointestinal diseases as the outcome, while the blue lines and rectangles represent the reverse analysis with chronic gastrointestinal diseases as the exposure and LTL as the outcome. (**B**) The MR analysis relies on three key assumptions

A concise flowchart illustrating a two-sample bidirectional Mendelian randomization analysis of LTL and four chronic digestive diseases is depicted in Fig. 1A. This study utilized summary-level GWAS data an exposure factor to evaluate its impact on the four diseases. Conversely, the reverse MR analysis employed the four diseases as exposure factors to assess potential reverse causal effects on LTL. The three indispensable key assumptions of Mendelian randomization, forming the foundation for all analyses in this study, are illustrated in Fig. 1B. This study was conducted following the guidelines of Strengthening the Reporting of Observational Studies in Epidemiology using Mendelian Randomisation (STROBE-MR) [15, 16].

### Data source

All Summary-level GWAS data from European populations were obtained from the MRC Integrative Epidemiology Unit (IEU, https://gwas.mrcieu.ac.uk) [17, 18], with specific cases and controls shown in Fig. 1A and **Table ****S1**.

Data for LTL from the UK Biobank including 472,174 individuals was downloaded from IEU (GWAS ID: IEU-b-4879). LTL was estimated using a quantitative PCR-based method by measuring the relative copy number ratio of telomere sequences to single-copy gene sequences [19]. LTL- GWAS was adjusted for covariates such as sex, age, ethnicity, and technical factors [19].

Data for IBS from the UK Biobank and Bellygenes initiative including 486,601 individuals (53400cases/ 433201controls) was downloaded from IEU (GWAS ID: ebi-a-GCST90016564).All patients with IBS meet at least one of the following four conditions: [1] fulfill the Rome III symptom criteria for the diagnosis of IBS, with no other symptoms or signs confirming another disease; [2] self-acknowledge a diagnosis of IBS; [3] self-report symptoms consistent with the diagnosis of IBS; [4] clinically diagnosed with IBS by a clinical physician based on ICD-10 criteria [20]. IBS-GWAS data was adjusted for covariates such as sex, age and the first 20 genetic principal components [20].

Data for PUD from the UK Biobank-Neale lab consortium including 361,194 individuals (3467 cases/ 357727controls) was downloaded from IEU (GWAS ID: ukb-d-K11_GASTRODUOULC). Diagnosis of PUD in the Neale lab consortium follows ICD-10 criteria. PUD-GWAS data was adjusted for covariates such as age, gender, genotyping array, and the first 20 genetic principal components. Further information can be found on the official website (http://www.nealelab.is/uk-biobank).

Data for GERD from the FinnGen including 202,836 individuals (13141cases/ 189,695 controls) was downloaded from IEU (GWAS ID: finn-b-K11_REFLUX). Data for NAFLD from the FinnGen including 218,792 individuals (894cases/ 217,898 controls) was downloaded from IEU (GWAS ID: finn-b-NAFLD). Diagnosis of related diseases in FinnGen follows ICD-10 criteria. The FinnGen-GWAS data was adjusted for covariates such as sex, age, genotyping array, the first ten genetic principal components, and genetic related matrix. Further information can be found on the official website (https://www.finngen.fi/en)[21].

All data utilized in this study were sourced openly, and their ethical reviews were explicitly declared in the original studies and biobanks. Therefore, there is no need for an additional ethical assessment in the context of this study.

### Instrumental variables selection

Qualified SNPs were selected from the exposure-outcome GWAS summary-level data as instrumental variables (IVs) for MR analysis.

#### Forward MR analysis

In the forward MR analysis, SNPs meeting three criteria were selected as IVs: initially, SNPs with a genome-wide significance threshold of p1 < 5 × 10^− 8^. Subsequently, to eliminate potential linkage imbalance effects, SNPs with an r2 > 0.001 of the most significant SNPs within the range of 10,000 kb were excluded. Lastly, SNPs highly correlated with the outcome were excluded, utilizing a threshold of p2 < 5 × 10^− 8^. Before harmonization with the outcome, a total of 154 valid SNPs that could serve as alternatives to LTL were identified. For accuracy and consistency in selecting SNPs as qualified IVs for different analyses, we refrain from seeking proxies for missing SNPs. Additionally, palindromic SNPs with intermediate allele frequencies (MAF = 0.5) were removed during harmonization of exposure and outcome data.Furthermore, we reviewed the literature to identify established factors influencing the outcome and used the R package “phenoscanner” and GWAS catalog (http://www.ebi.ac.uk/gwas) to eliminate potential confounding factors SNPs, the effect p value was selected as 1 × 10^− 5^ [22, 23]. Before harmonization with IBS, we excluded the effects of BMI, smoking, alcohol consumption, and mental health factors [24, 25]. Before harmonization with GERD, we excluded the effects of BMI, weight, and diabetes [26]. Before harmonization with PUD, we excluded the effects of smoking, alcohol consumption, mental health, and sleep factors [27, 28]. Before harmonization with NAFLD, we excluded the effects of BMI, mental health, smoking, diabetes, and sleep factors [29, 30]. To avoid false negatives caused by weak instrument variables, we calculate the F-statistic for each SNP in the exposure factor, ensuring that all the F-values are greater than 10 (Table S2–9) [F = R^2^(N − K−1)/K(1 − R^2^), R^2^ = 2×EAF× (1 − EAF) ×β] [13, 14]. Ultimately, we filtered out 116, 136, 126, and 120 valid instrumental variables, respectively, for subsequent forward MR analyses targeting IBS, GERD, PUD, and NAFLD (Table S2–5).

#### Reverse MR analysis

In the reverse MR analysis, due to potential possible reasons such as small sample size and shallow sequencing depth, the number of SNPs reflecting disease exposure extracted when the genome-wide significance threshold of SNPs was set to p1 < 5 × 10^− 8^ was small, so we set it to p1 < 5 × 10^− 6^. We applied all discovered IVs to the reverse MR analysis. Furthermore, the remaining criteria for filtering IVs were the same as those used in the forward MR analysis. Ultimately, we filtered out 42, 24, 13, and 17 valid IVs, respectively, for subsequent reverse MR analyses targeting LTL in the IBS, GERD, PUD, and NAFLD cohorts (Supplementary File 2 – Table S6–9).

### MR analysis statistical analysis

#### MR analysis

To assess the potential bidirectional causal relationship between LTL and the four diseases, we utilized inverse-variance weighted (IVW) as the primary method for MR analysis. Supplementary analyses were conducted using methods such as MR-Egger regression, weighted-median, Maximum Likelihood (MaxLik), Robust adjusted profile score (MR-RAPS), Contamination mixture (ConMix), and MR-mix. The IVW method initiates by using Wald estimator and the Delta method to compute individual SNP ratio estimates. These estimates are then aggregated to derive the primary causal estimate. Operating within the parameters of satisfying the three foundational assumptions of Mendelian Randomization analysis and steering clear of weak instrument bias, IVW method emerges as a robust technique in MR analysis [31].To evaluate heterogeneity among the chosen SNPs, we employ Cochran’s Q test. In instances of heterogeneity (*p* < 0.05), the random-effects IVW method is utilized; otherwise, the fixed-effects IVW method is utilized [32]. Acknowledging that the outcomes of the IVW method may be influenced by effective instruments, potential pleiotropic effects, and sample overlap, we enhance our analysis with other MR methods. For a more comprehensive exploration of additional MR methods, please refer to Supplementary File 1.

#### Sensitivity analysis

To validate the study findings, a series of sensitivity tests were conducted. The intercept and p-value of the MR-Egger regression curve were examined to evaluate the impact of directional pleiotropy, with an intercept approaching 0 and *p* > 0.05 indicating negligible bias [33, 34]. MR-PRESSO was applied to identify outlier SNPs and assess their potential horizontal pleiotropy [35]. leave-one-out analysis was conducted to assess the influence of individual SNPs on the primary causal relationships [33]. After the removal of outlier SNPs, Cochran’s Q statistic and a funnel plot were employed to detect heterogeneity in the IVW and MR Egger regression methods [31].

For binary outcome variables, the odds ratio (OR), 95% confidence interval (CI), and p-value were employed to quantify the strength of the causal relationship. Regarding continuous outcome variables, β, standard error (SE), and p-value were utilized to gauge the magnitude of the causal effect. Statistical power for the MR analysis wascomputed using an online tool [36] (https://shiny.cnsgenomics.com/mRnd/). The bias resulting from sample overlap was assessed using another online tool (https://sb452.shinyapps.io/overlap/). The MR analysis was carried out using R software version 4.2.2.

## Result

### Causal effects of LTL on digestive system diseases

**Fig. 2:**
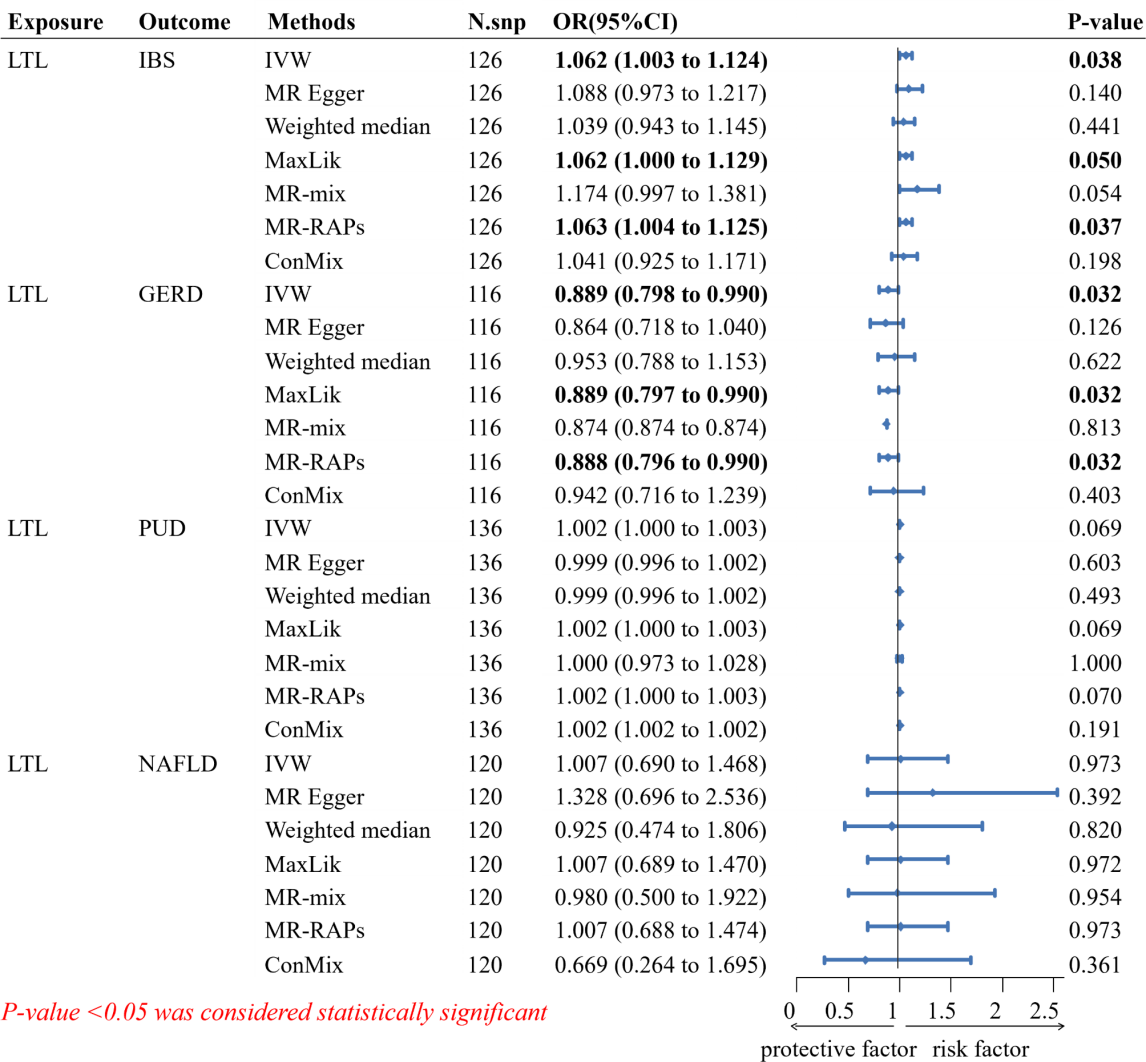
Forest plot of the associations between genetically determined LTL with the risk of gastrointestinal diseases. Abbreviation: LTL, leukocyte telomere length. IBS, irritable bowel syndrome. GERD, gastroesophageal reflux disease. PUD, peptic ulcers disease. NAFLD, nonalcoholic fatty liver disease. OR, Odds ratio. CI, Confdence interval. N.snp, the number of single nucleotide polymorphisms included in the Mendelian randomization analysis. IVW, Inverse-variance weighted. ConMix, Contamination mixture. MR-RAPS, Robust adjusted profile score

For IBS, it was positively associated with the risk of LTL in IVW method (OR: 1.062; CI: 1.003, 1.124; *p* = 0.038, power = 0.67). The MaxLik method (OR: 1.062; CI: 1.000, 1.129; *p* = 0.050, power = 0.67) and the MR-RAPs method (OR: 1.063; CI: 1.004, 1.125; *p* = 0.037, power = 0.63) also supported this result **(**Fig. 2**)**. The MR-PRESSO test and leave-one-out analysis did not reveal clear outlier IVs. The intercept of the MR-Egger regression also indicated no horizontal pleiotropy (*p* = 0.61). There was no significant heterogeneity among the IVs by Cochran’s Q test based on IVW (*p* = 0.15), and MR-Egger regression (*p* = 0.14) **(**Table 1**)**.

**Table 1:** Results of sensitivity tests for forward Mendelian randomization analysis

Exposure	Outcome	N.snp	heterogeneity				pleiotropy			
			MR Egger		IVW		MR-Egger regression		MR-PRESSO	
			Q	*p*-value	Q	*p*-value	Intercept	*p*-value	RSSobs	*p*-value
LTL	IBS	126	141.15	0.14	141.45	0.15	0.00	0.61	143.92	0.15
LTL	GERD	116	113.81	0.49	113.94	0.51	0.00	0.72	115.84	0.51
LTL	PUL	136	100.75	0.99	104.34	0.98	0.00	0.06	106.59	0.97
LTL	NAFLD	120	100.81	0.87	101.88	0.87	-0.01	0.30	104.32	0.88

For GERD, it was positively associated with the risk of LTL in IVW method (OR: 0.889; CI: 0.798, 0.990; *p* = 0.032, power = 0.92). The MaxLik method (OR: 0.889; CI: 0.797, 0.990; *p* = 0.032, power = 0.92) and the MR-RAPs method (OR: 0.888; CI: 0.796, 0.990; *p* = 0.032, power = 0.92) also supported this result **(**Fig. 2**)**. The MR-PRESSO test and leave-one-out analysis did not reveal clear outlier IVs. The intercept of the MR-Egger regression also indicated no horizontal pleiotropy (*p* = 0.72). There was no significant heterogeneity among the IVs by Cochran’s Q test based on IVW (*p* = 0.51), and MR-Egger regression (*p* = 0.49) **(**Table 1**)**.

For PUD and NAFLD, no statistically significant associations were found between LTL and these diseases in all MR analyses (Fig. 2). Although the results of sensitivity tests indicated no statistically significant heterogeneity and horizontal pleiotropy among IVs, the statistical power of the MR analysis was notably low (range: 0.05–0.42), potentially indicating compromised robustness of the results, likely influenced by sample size limitations (Table 1).

The F-values of the 154 effective IVs associated with the LTL are all greater than 10, ranging from 29.9 to 1628.8. Therefore, the likelihood of the forward MR analysis being influenced by weak instrumental variables is relatively low. All forwardMR analysis results are presented in Fig. 2 and Supplementary Figs. 1–4. Detailed data used for the forward MR analysis of LTL on IBS, GERD, PUD, and NAFLD can be found in the Table S2, 3, 4, 5.

The statistical power of the forward MR analysis is presented in Table S10. The MR analysis results of LTL in relation to IBS and GERD demonstrate ideal statistical power. However, due to the limited number of case samples, the MR analysis results for LTL in relation to PUD and NAFLD exhibit very low statistical power. The impact of sample overlap on the statistical error of MR analysis is illustrated in Table S12. The MR analysis results for LTL in relation to IBS show no significant bias in the overlap range of 0–20%, and there is no substantial increase in the probability of Type I error with 100% sample overlap. The MR analysis results for LTL in relation to PUD show no significant bias in the overlap range of 0–10%, but when the sample overlap rate reaches 60%, the probability of Type I error will rise to 6%.

### Causal effects of digestive system diseases on LTL

To assess whether the four diseases have an impact on LTL, we conducted reverse MR analyses. Among all the analyses, only the MR analysis of IBS on LTL based on the MR-RAPs method showed statistical significance (β: -0.019, SE: 0.009, *p* = 0.049). In contrast, the results of MR analyses based on IVW indicated that none of these four diseases had a statistically significant impact on LTL (β: -0.018, SE: 0.010, *p* = 0.093 for IBS; β: 0.004, SE: 0.007, *p* = 0.557 for GERD; β: 0.730, SE: 0.501, *p* = 0.145 for PUD; β: 0.000, SE: 0.002, *p* = 0.973 for NAFLD) (Table 2).

**Table 2:** Results of sensitivity tests for reverse Mendelian randomization analysis

Exposure	Outcome	N.snp	heterogeneity				pleiotropy			
			MR Egger		IVW		MR-Egger regression		MR-PRESSO	
			Q	*p*-value	Q	*p*-value	Intercept	*p*-value	RSSobs	*p*-value
IBS	LTL	42	52.64	0.09	54.85	0.07	0.00	0.20	57.53	0.08
GERD	LTL	24	26.65	0.22	26.87	0.26	0.00	0.68	29.10	0.28
PUL	LTL	13	9.45	0.58	9.49	0.66	0.00	0.84	11.14	0.68
NAFLD	LTL	17	12.64	0.63	13.37	0.65	0.00	0.40	15.03	0.68

In the analysis of the relationship between GERD and LTL, MR-PRESSO test detected 3 outlier SNPs (rs 10,112,752, rs117630647 and rs10805346), which were subsequently excluded from the analysis. In the MR analysis of LTL on the other three diseases, both the MR-PRESSO test and leave-one-out analysis did not identify any clear outlier IVs. Furthermore, sensitivity tests across all analyses did not indicate heterogeneity or horizontal pleiotropy among the included instrumental variables **(**Table 2**)**. All reverse MR results are presented in Table 3 and Supplementary Figs. 5–8. Detailed data used for the reverse MR analysis of LTL on IBS, GERD, PUD, and NAFLD can be found in the Table S6, 7, 8, 9.

**Table 3:** Result of the associations between genetically determined gastrointestinal diseases with the risk of LTL.

Exposure	Outcome	Methods	N.snp	β	se	*p*-value
IBS	LTL	IVW	42	-0.018	0.010	0.093
		MR Egger	42	0.025	0.035	0.470
		Weighted median	42	0.003	0.013	0.836
		MaxLik	42	-0.018	0.011	0.098
		MR-mix	42	-0.040	0.093	0.667
		MR-RAPs	42	**-0.019**	**0.009**	**0.049**
		ConMix	42	-0.010	0.010	0.317
GERD		IVW	24	0.004	0.007	0.557
		MR Egger	24	0.010	0.015	0.535
		Weighted median	24	0.003	0.009	0.742
		MaxLik	24	0.004	0.007	0.552
		MR-mix	24	-0.010	0.026	0.701
		MR-RAPs	24	0.004	0.006	0.524
		ConMix	24	0.014	0.008	0.431
PUD		IVW	13	0.730	0.501	0.145
		MR Egger	13	0.903	0.992	0.382
		Weighted median	13	0.504	0.676	0.456
		MaxLik	13	0.760	0.511	0.137
		MR-mix	13	0.730	34.546	0.983
		MR-RAPs	13	0.753	0.528	0.154
		ConMix	13	1.739	1.270	0.153
NAFLD		IVW	17	0.000	0.002	0.973
		MR Egger	17	-0.002	0.003	0.507
		Weighted median	17	-0.001	0.002	0.523
		MaxLik	17	0.000	0.002	0.973
		MR-mix	17	0.000	0.021	1.000
		MR-RAPs	17	0.000	0.002	0.974
		ConMix	17	0.000	0.000	0.906

The statistical power of the reverse MR analysis is presented in Table S11. The MR analysis results for IBS, GERD, and PUD in relation to LTL demonstrate ideal statistical power, while the results for NAFLD exhibit very low statistical power. The impact of sample overlap on the statistical error of MR analysis is shown in Table S12. The analysis results for IBS in relation to LTL show no significant bias in the overlap range of 0–10%, but when sample overlap rate reaches 50%, the probability of Type I error will rise to 6%. The MR analysis results for PUD in relation to LTL show no significant bias in the overlap range of 0–20%, and even with 100% sample overlap, the probability of Type I error does not increase.

## Discussion

Telomere length serves as a widely recognized biological indicator of cellular aging and is intricately connected to various age-related ailments, including coronary heart disease and diabetes [1–3]. To our knowledge, this study represents the first attempt to explore the relationship between LTL and four digestive diseases (IBS, GERD, PUD, and NAFLD) using a Mendelian randomization (MR) study. Our research indicates that, when accounting for potential confounding factors and reverse causation, three MR analysis methods (IVW, MaxLik, and MR-RAPS) suggest a potential causal effect between LTL and IBS or GERD. Unfortunately, these findings were not corroborated by the other four MR analysis methods (MR-Egger regression, Weighted Median, ConMix, and MR-Mix). Therefore, our study falls short of fully confirming the causal relationship between LTL and IBS or LTL and GERD, but it highlights the potential significant role of LTL in digestive diseases. While none of the MR analyses demonstrated statistical significance in the association between LTL and PUD or LTL and NAFLD, limitations in statistical power due to the small sample sizes cannot entirely rule out causal relationships between LTL and PUD or LTL and NAFLD.

As previously mentioned, Zhang. et al. studies indicated a correlation between shorter telomere length (TL) and a higher incidence of IBS [8]. However, it’s important to note that a substantial number of patients with a history of depression, an important condition well-established to lead to telomere shortening [37, 38], were included in these studies. Psychological factors as potential confounding factors may contribute to some outcome bias. In our study, after excluding the interference of SNPs associated with psychological traits, we obtained the opposite results from previous studies, which may be related to the fact that mental health is associated with IBS. This aligns with epidemiological statistics for IBS, which predominantly affects young and middle-aged women, showing an inverse relationship between telomere length and age. Souza et al. reported shorter TL in the esophageal tissues of GERD patients, showing no variation with age [9]. In contrast, our study measured telomere length in circulating leukocytes, and previous research has shown that LTL can serve as a proxy for telomere length in other tissue types [5], thus providing further validation for this conclusion.

Concerning the association between telomere length and NAFLD incidence, consensus has not been reached. While some studies indicate a correlation between telomere shortening and NAFLD, numerous complex confounding factors, including population ethnicity, age, and diabetes, come into play [10–12]. Despite our study’s efforts to control for these confounding factors, we obtained negative results between LTL and NAFLD. Additionally, no association between PUD and LTL was identified. However, it’s crucial to note that when assessing the relationship between LTL and PUD or NAFLD, the MR analysis exhibited extremely low statistical power due to the limited number of case samples. This may suggest that, at this stage, MR analysis might not be suitable for inferring causal effects between LTL and PUD or LTL and NAFLD.

We attempted to explore the relationship between LTL and IBS, or LTL and GERD, considering the potential influence of the brain-gut axis. The brain-gut axis denotes a bidirectional communication system between the brain and the digestive system, regulating functions through neural, endocrine, and immune pathways [39]. Telomerase, vital for maintaining telomere length and cell stability during cell division, can regenerate missing telomere sequences [9, 10]. In young populations, higher telomerase activity counteracts telomere shortening, supporting normal biological function. The presence of the brain-gut axis may be linked to the occurrence of certain gastrointestinal diseases. slowly cycling gastrointestinal cells could generate an inhibitory signal suppressing the brain-gut axis. Upon recognition by the central nervous system, this signal triggers a negative feedback effect, releasing stress hormones through the sympathetic nervous system, thereby triggering gastrointestinal hyperactivity, and increasing gastrointestinal sensitivity [39], leading to symptoms associated with IBS. Conversely, as telomerase activity diminishes, LTL shortens with accelerated cell division. The brain-gut axis recognizes potential afferent signals, feedback-exciting vagus nerve excitation, inhibiting esophageal-gastric sphincter contraction, and triggering gastroesophageal reflux. Telomeres, identified as the preferred effector site of DNA oxidation [40], are prone to chronic inflammation due to gastrointestinal damage, leading to the accumulation of reactive oxygen species and subsequent oxidative damage to DNA telomeres, resulting in LTL shortening [41]. This forms a vicious circle, leading to abnormal regeneration after esophageal squamous epithelial injury, cell metamorphosis, and eventually, the development of cancer.

Moreover, it is crucial to acknowledge that mental state and life stress exert diverse influences on LTL and digestive system diseases. Research has demonstrated that depression, a fast-paced lifestyle, and disruptions in biorhythms impact LTL [37, 38]. This implies a complex interrelation between the onset of digestive system diseases and LTL, indicating a complex correlation rather than a straightforward causal association. Nevertheless, our study implies that the assessment of LTL could evolve into a biomarker for gauging the risk factors associated with intestinal diseases.

The strength of our study lies in its pioneering use of Mendelian randomization analysis to investigate the association between LTL and four chronic digestive system diseases, aiding in the reduction of bias arising from reverse causation and confounding factors. Nevertheless, our study grapples with certain challenging limitations. Firstly, the limited number of cases restricts the statistical power of the MR analysis for LTL and its correlation with PUD and NAFLD which indicates that we cannot conclusively rule out the presence of causation between them. Secondly, the GWAS summary-level data for LTL, IBS, and PUD are all derived from the UK Biobank. While the likelihood of bias from sample overlap is relatively small, it remains an inevitable risk factor. Thirdly, the incomplete similarity in linkage disequilibrium patterns between the Finnish and UK Biobank GWAS data may introduce bias to the study. Lastly, our research exclusively involves individuals of European ethnicity, potentially limiting the universal applicability of the study’s conclusions to other ethnic groups.

## Conclusion

The current evidence does not definitively rule out a causal relationship between LTL and these four gastrointestinal diseases but suggests a potential association LTL and IBS, or LTL and GERD. Exploring the relationship between gastrointestinal diseases may offer new insights into the onset, progression, and treatment of these diseases.

### Electronic supplementary material

Below is the link to the electronic supplementary material.

Supplementary Material 1

Supplementary Material 2

Supplementary Material 3

Supplementary Material 4

## Data Availability

The datasets are available in the MRC Integrative Epidemiology Unit (IEU, https://gwas.mrcieu.ac.uk). Please refer to the supplementary materials for additional data related to this study.

## List of abbreviations

LTL: Leukocyte telomere length
IBS: Irritable bowel syndrome
GERD: Gastroesophageal reflux disease
PUD: Peptic ulcers disease
NAFLD: Nonalcoholic fatty liver disease
GWAS: Genome-wide association study
CI: Confdence interval
IV: Instrumental variable
IVW: Inverse-variance weighted method
LD: Linkage disequilibrium
MR: Mendelian randomization
MR-PRESSO: MR pleiotropy residual sum and outlier
ConMix: Contamination mixture
MR-RAPS: Robust adjusted profile score
OR: Odds ratio
SNP: Single nucleotide polymorphisms
LD: Linkage disequilibrium
MAF: Minor allele frequency
EAF: Effect Allele Frequency
