# Supplementary material for: Exploration of the causal effects of leukocyte telomere length and four gastrointestinal diseases: a two-sample bidirectional Mendelian randomization study
Source: BMC Gastroenterol. 2023 Dec 18;23:446. doi: 10.1186/s12876-023-03081-y (PMC10729385; doi:10.1186/s12876-023-03081-y)
Supplement: Supplementary file 1 — Supplementary Material 1 [file 12876_2023_3081_MOESM1_ESM.docx]

## ****Supplementary MR analysis methods****

### MR-Egger regression

The MR-Egger method assumes that the distribution of the direct impact of candidate IVs on the outcome is independent of the distribution associated with the exposure factor, referred to as the "Instrument Strength Independent of Direct Effect (InSIDE)" assumption[1]. This is achieved through a regression analysis of the precision-weighted IVs estimates related to the exposure factor against the outcomes associated with the IVs. The intercept of the regression can be interpreted as an estimate of the average pleiotropic effect of the IVs. Even when all Ivs are ineffective due to a violation of the third assumption of MR analysis, the regression slope can still provide an estimate of the causal effect.

### Weighted Median

The weighted median method ranks MR estimates for each IV based on its precision-weighted magnitude and generates an overall MR estimate using the median value[2]. It can offer a robust causal relationship analysis even in situations where up to 50% of instrumental variables are invalid.

### Maximum Likelihood

The Maximum Likelihood (MaxLik) method serves as a complement to the IVW approach, assuming conditions of no heterogeneity and horizontal pleiotropy in MR analysis[3]. If these assumptions hold, the results will be unbiased, and the standard errors will be smaller than those obtained from IVW. Furthermore, MaxLik can calculate MR estimates even in cases of complete sample overlap, offering additional support to the outcomes derived from the IVW method[4].

### Robust adjusted profile score

The Robust adjusted profile score (MR-RAPS) method builds a model for the pleiotropic effects of instrumental variables directly using a random-effects distribution. It assumes that the pleiotropic effects are centered around zero and follow a normal distribution with an unknown variance[5]. The estimate of the causal effect is obtained by fitting the profile-likelihood function to the variances of both the causal effect and pleiotropic effects distributions. MR-RAPS allows for the inclusion of some weak instrumental variables and provides robust statistical estimates for Mendelian Randomization by accounting for these weak instruments.

### Contamination mixture

The contamination mixture method (ConMix) is implemented by constructing a likelihood function based on variant-specific causal estimates.[6] For each IV, an estimate of the causal effect can be obtained by dividing the genetic association with the outcome by the genetic association with the exposure. ConMix categorizes instrumental variables into effective and ineffective groups. Under the assumption that there is a single causal effect of the exposure on the outcome, the method can robustly and efficiently estimate this effect, even when some genetic variants are not valid IVs.

### MRMix

The MR-Mix method adopts a modeling approach akin to ConMix[7]. However, it diverges by categorizing IVs into four groups: those exclusively affecting exposure (effective IVs), IVs impacting both exposure and outcome, IVs solely influencing the outcome, and IVs having no effect on either exposure or outcome (ineffective IVs). This provides greater flexibility in genetic variation modeling and serves as a reasonable complement to the ConMix method.

## ****Reference****

1. Bowden, J., G. Davey Smith, and S. Burgess, *Mendelian randomization with invalid instruments: effect estimation and bias detection through Egger regression.* Int J Epidemiol, 2015. **44**(2): p. 512-25.

2. Bowden, J., et al., *Consistent Estimation in Mendelian Randomization with Some Invalid Instruments Using a Weighted Median Estimator.* Genet Epidemiol, 2016. **40**(4): p. 304-14.

3. Pierce, B.L. and S. Burgess, *Efficient design for Mendelian randomization studies: subsample and 2-sample instrumental variable estimators.* Am J Epidemiol, 2013. **178**(7): p. 1177-84.

4. Yavorska, O.O. and S. Burgess, *MendelianRandomization: an R package for performing Mendelian randomization analyses using summarized data.* Int J Epidemiol, 2017. **46**(6): p. 1734-1739.

5. Slob, E.A.W. and S. Burgess, *A comparison of robust Mendelian randomization methods using summary data.* Genet Epidemiol, 2020. **44**(4): p. 313-329.

6. Burgess, S., et al., *A robust and efficient method for Mendelian randomization with hundreds of genetic variants.* Nat Commun, 2020. **11**(1): p. 376.

7. Qi, G. and N. Chatterjee, *Mendelian randomization analysis using mixture models for robust and efficient estimation of causal effects.* Nat Commun, 2019. **10**(1): p. 1941.
