# Supplementary material for: Exploration of the causal effects of leukocyte telomere length and four gastrointestinal diseases: a two-sample bidirectional Mendelian randomization study
Source: BMC Gastroenterol. 2023 Dec 18;23:446. doi: 10.1186/s12876-023-03081-y (PMC10729385; doi:10.1186/s12876-023-03081-y)
Supplement: Supplementary file 4 — Supplementary Material 4 [file 12876_2023_3081_MOESM4_ESM.pdf]

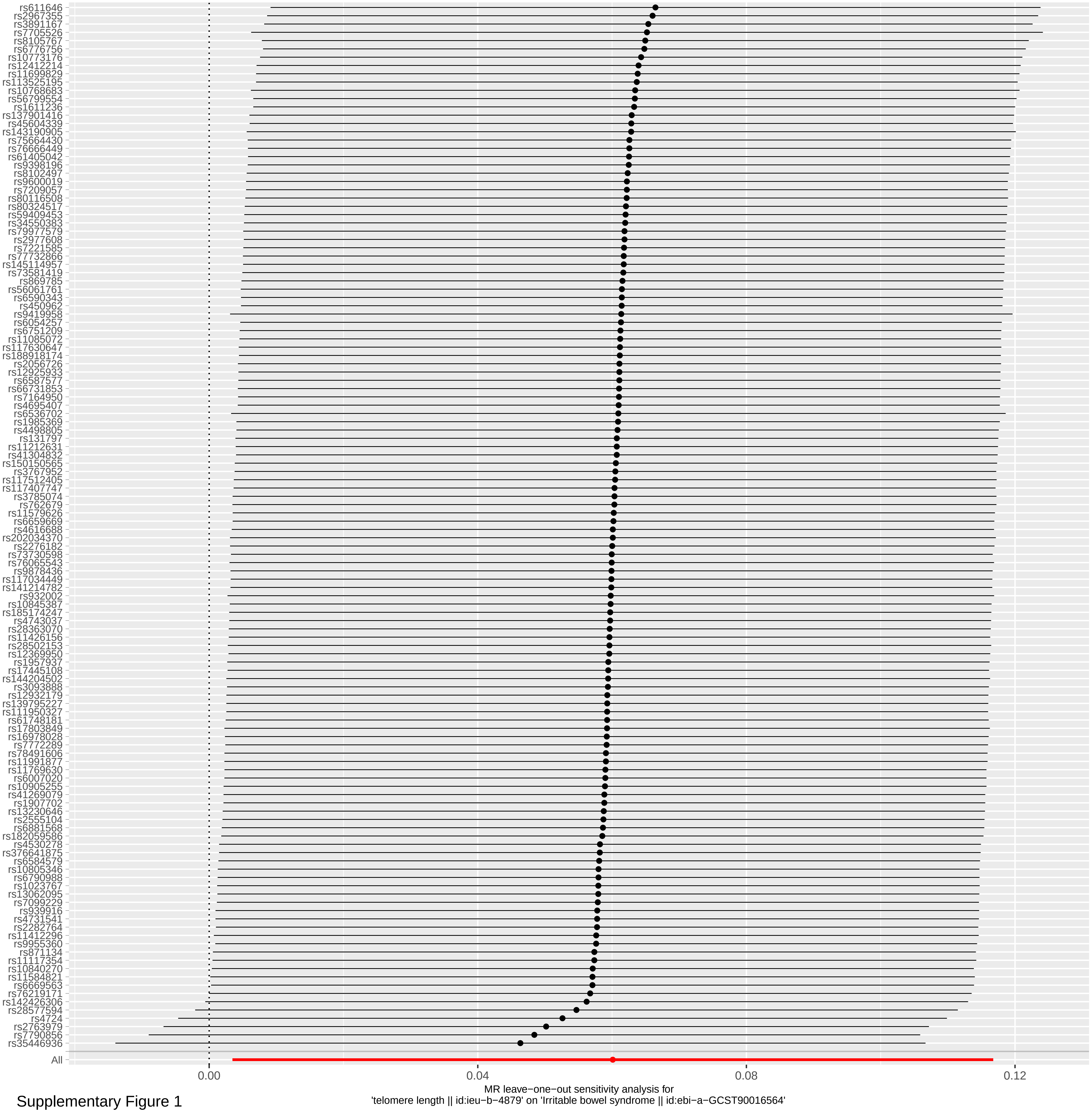

Supplementary Figure 1

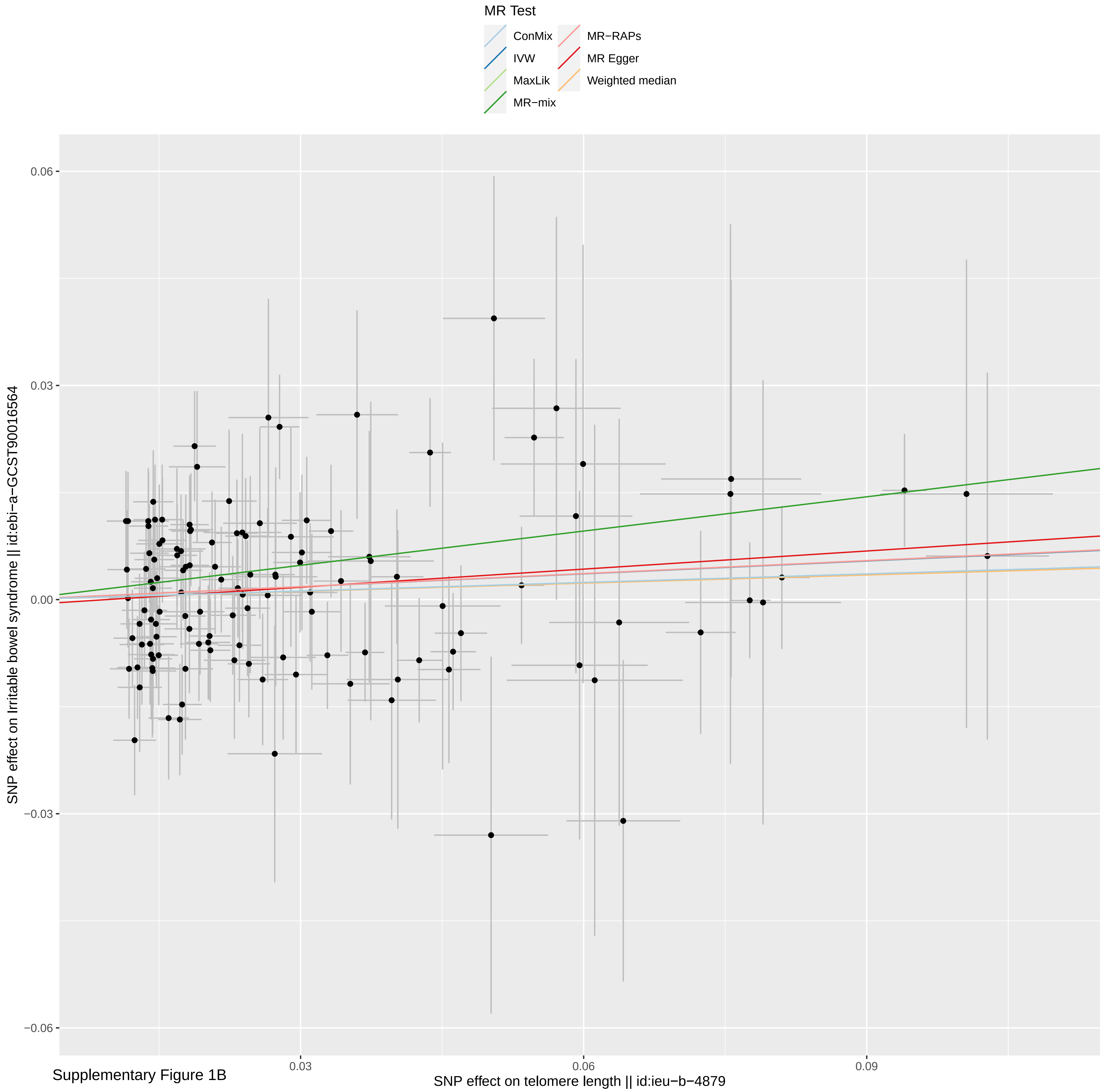

Supplementary Figure 1B

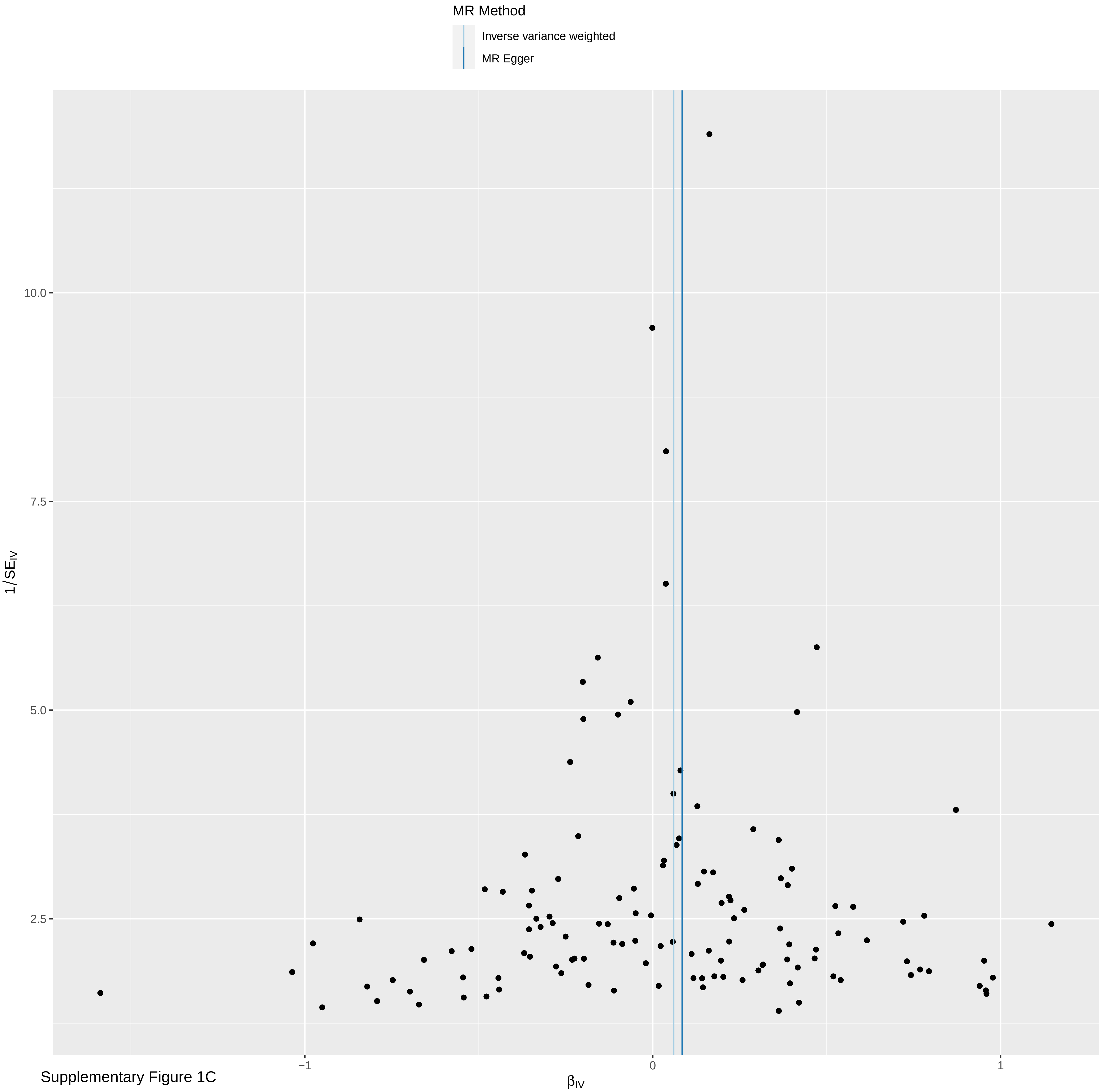

Supplementary Figure 1C

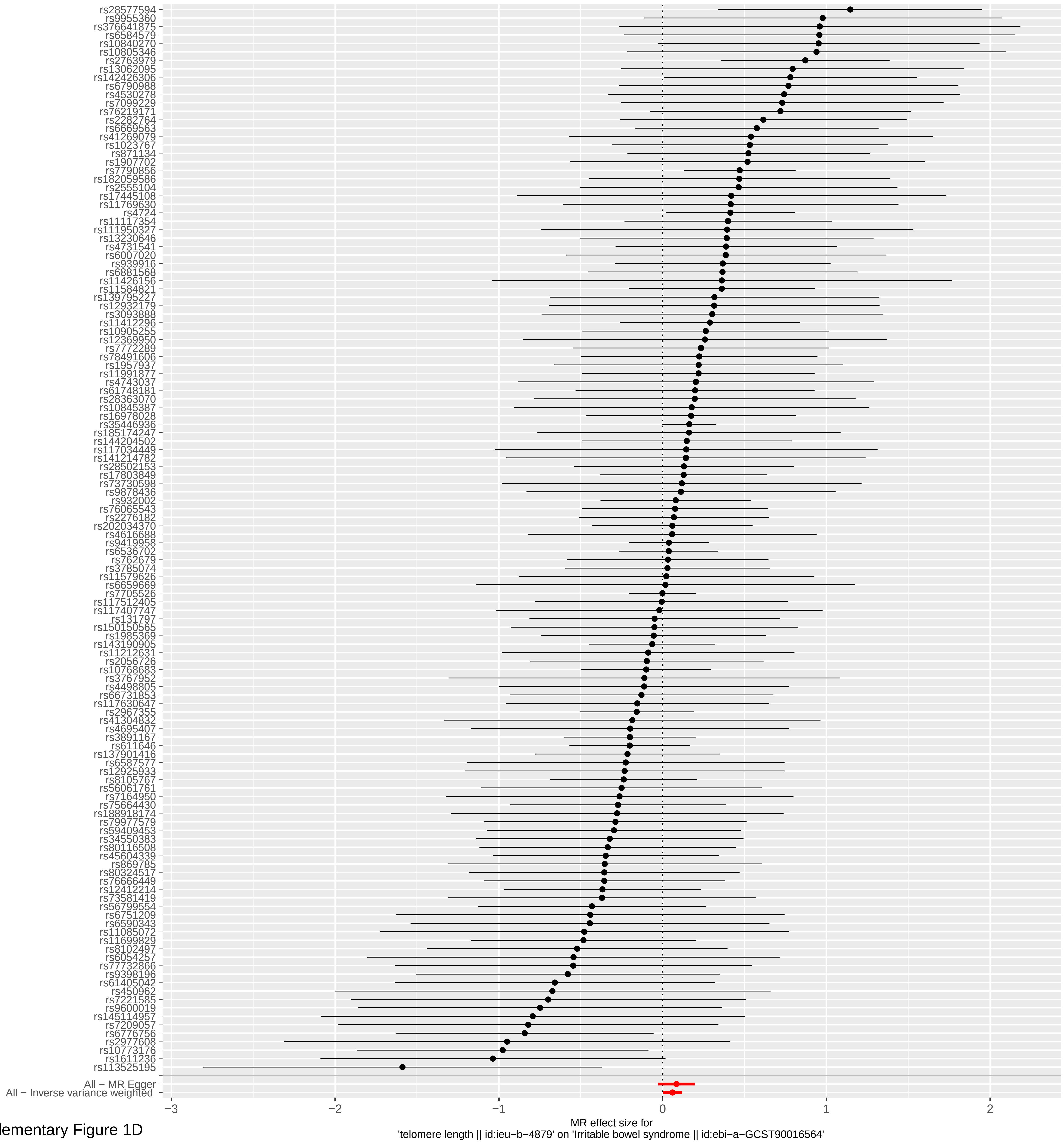

Supplementary Figure 1D

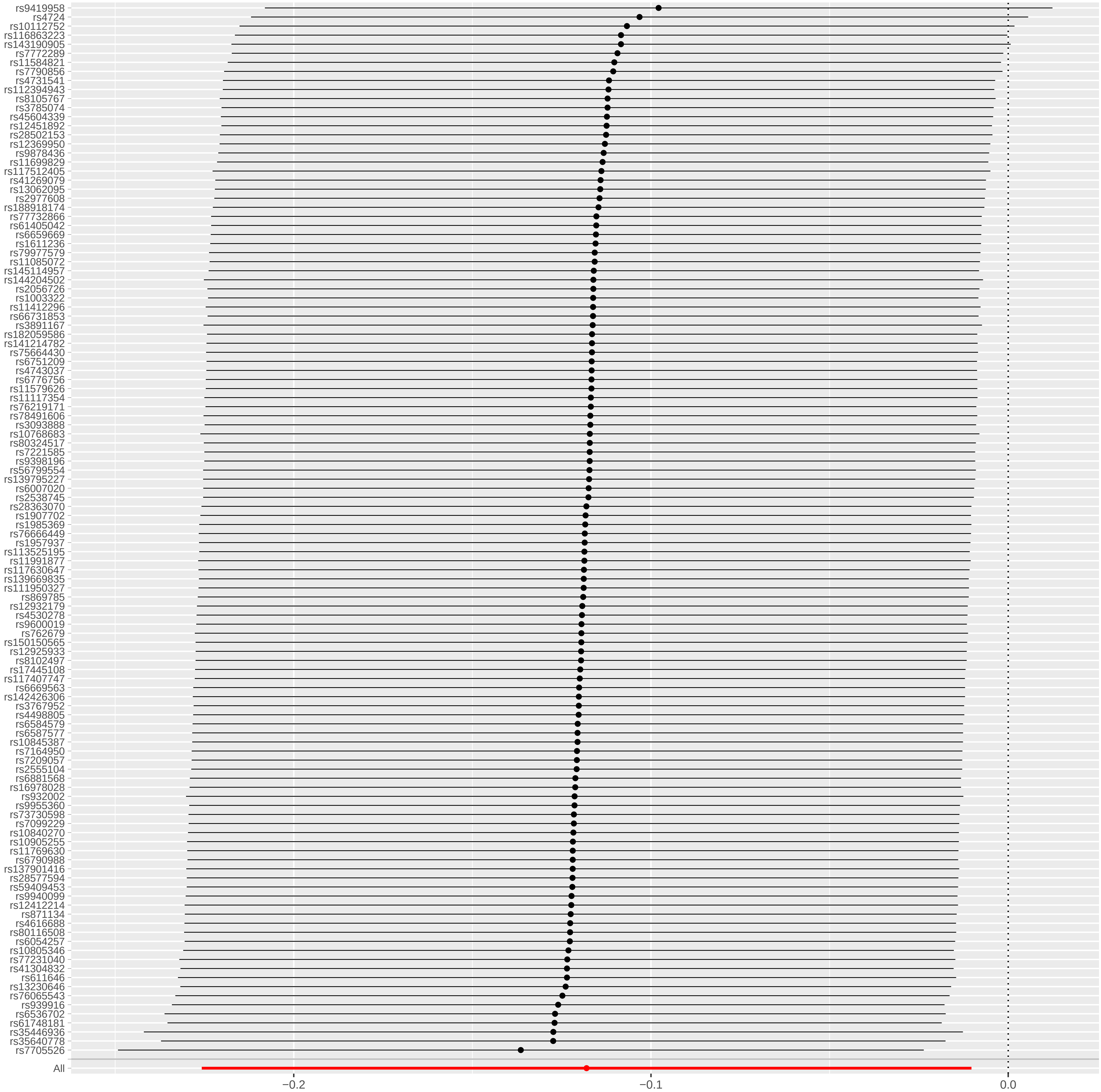

Supplementary Figure 2A

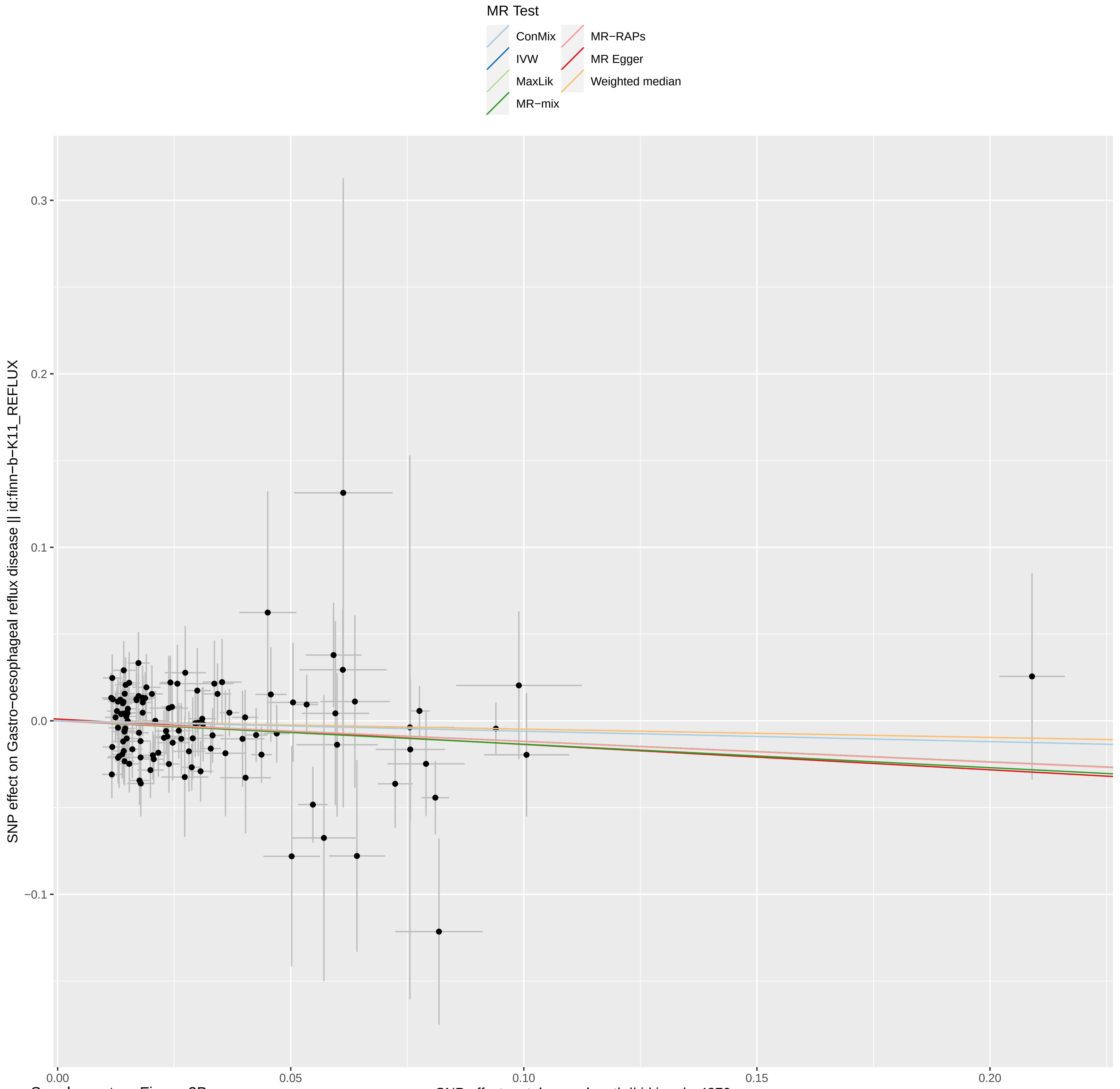

Supplementary Figure 2B

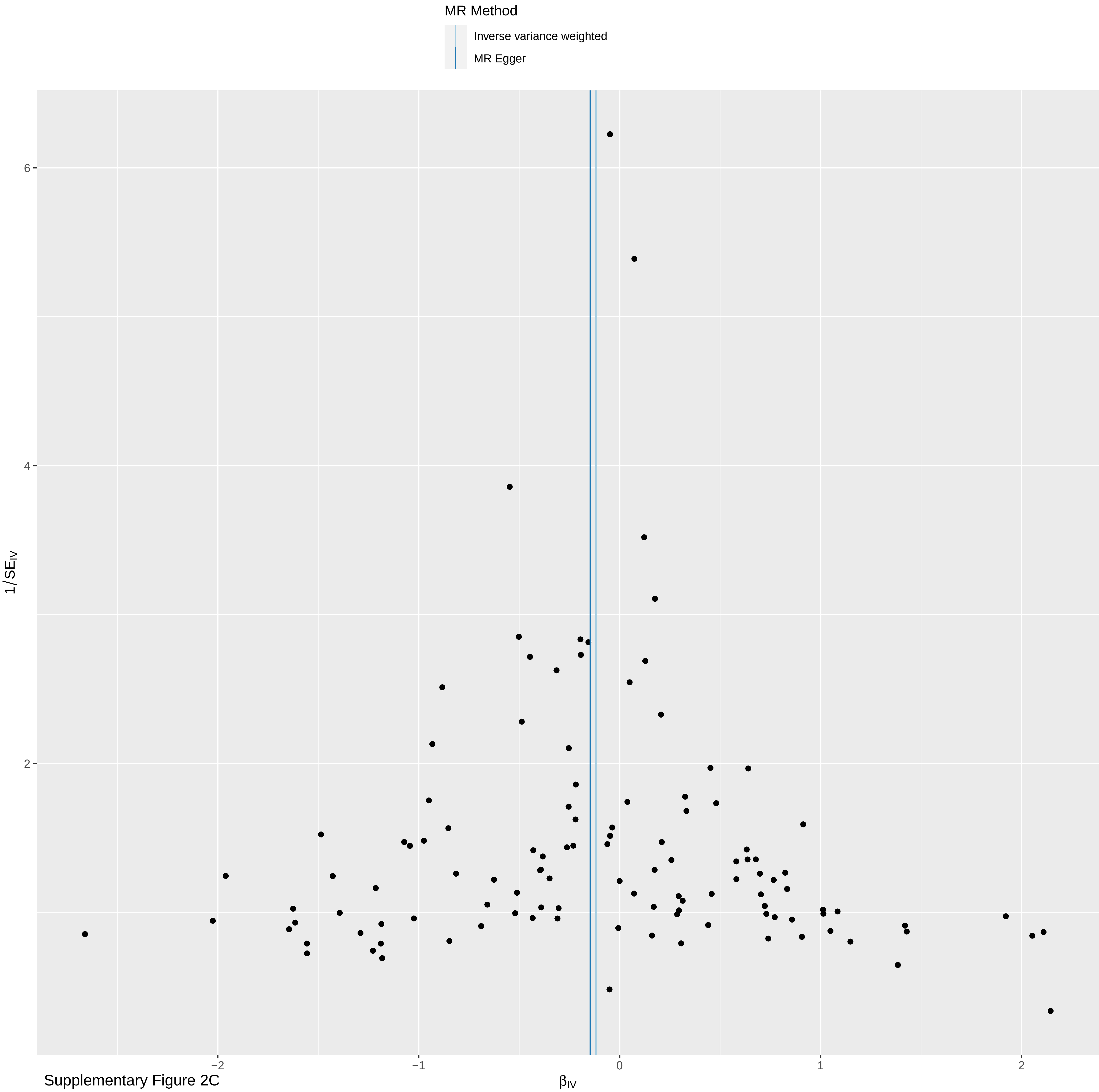

Supplementary Figure 2C

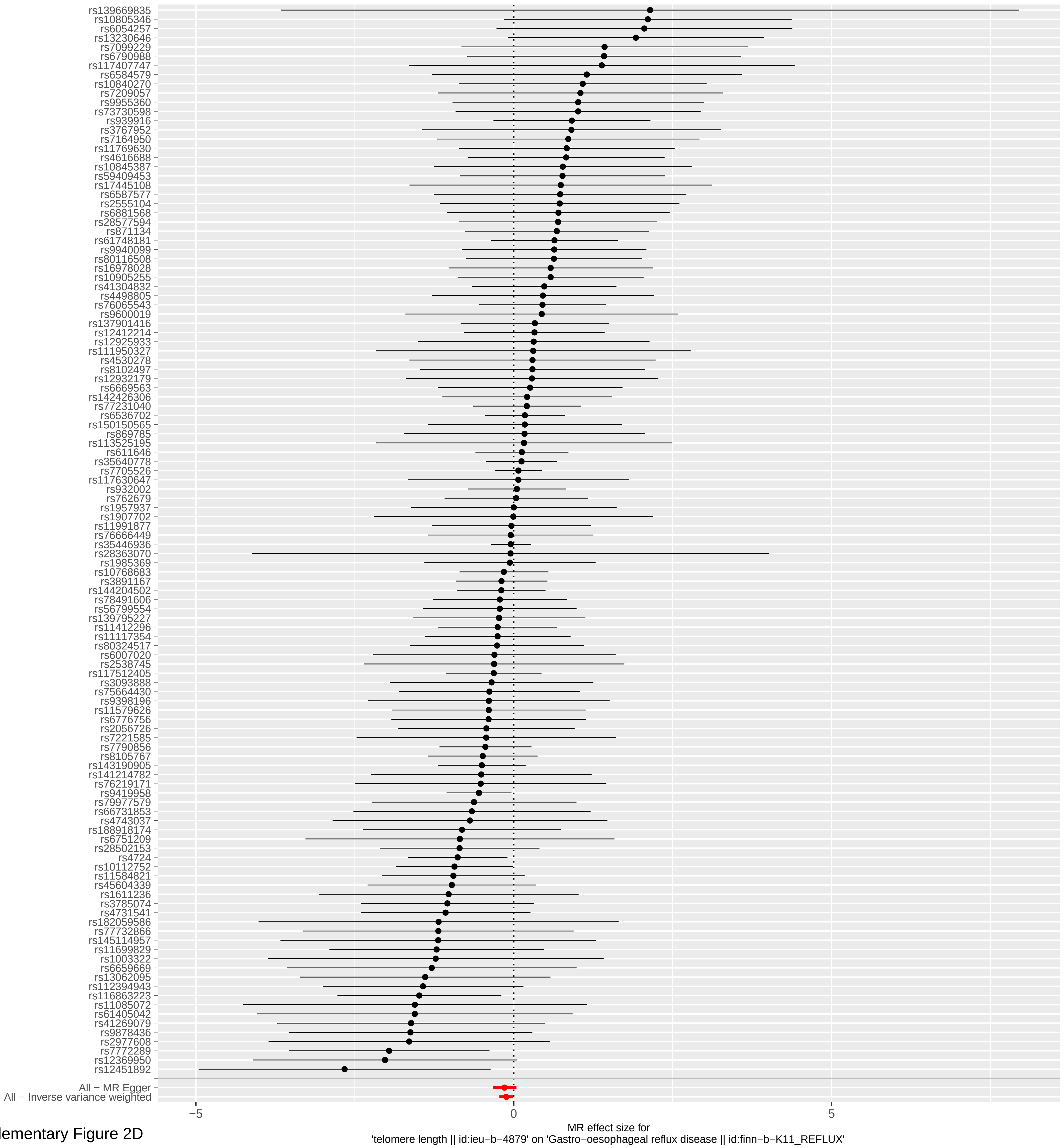

Supplementary Figure 2D

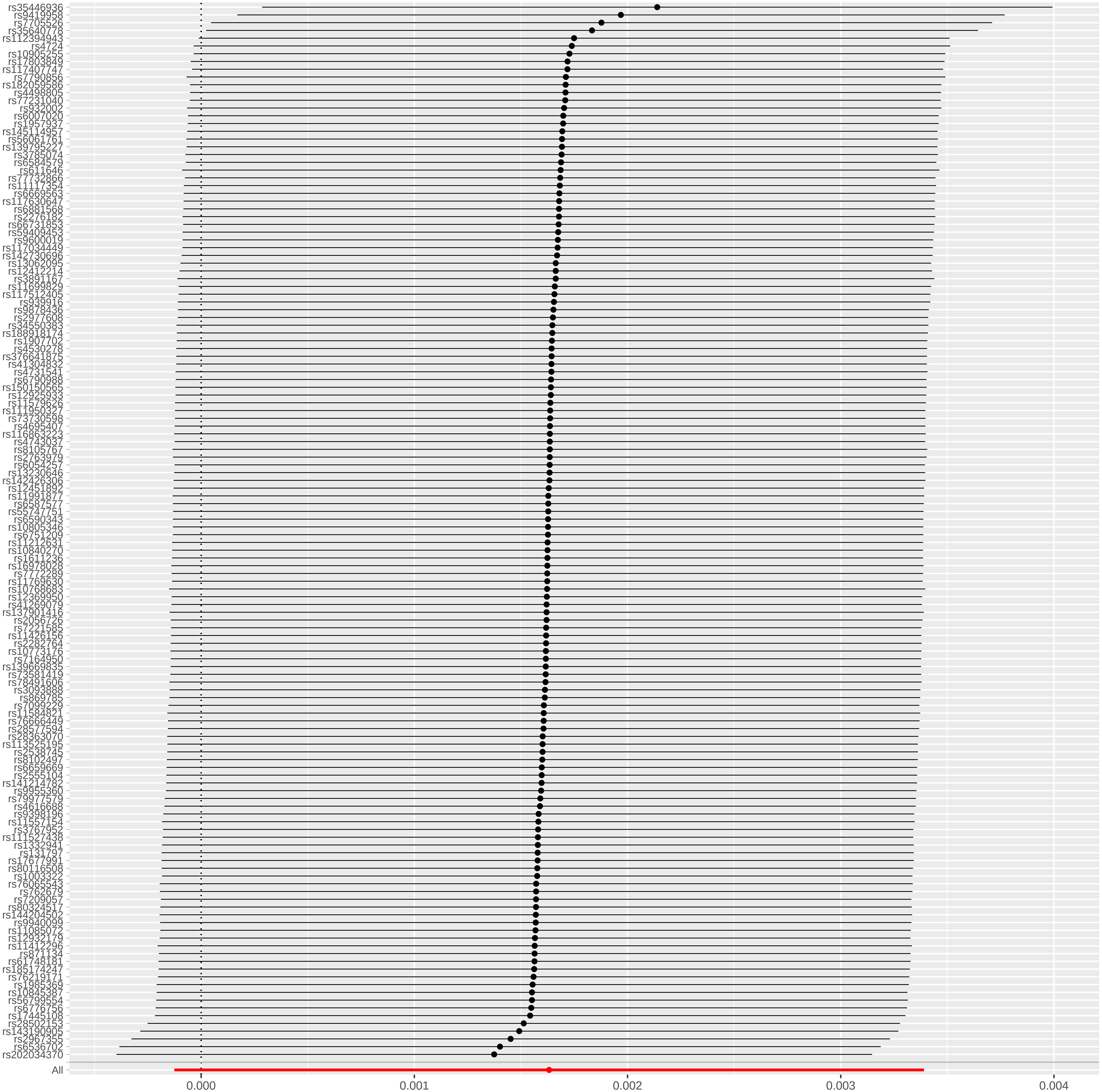

Supplementary Figure 3A

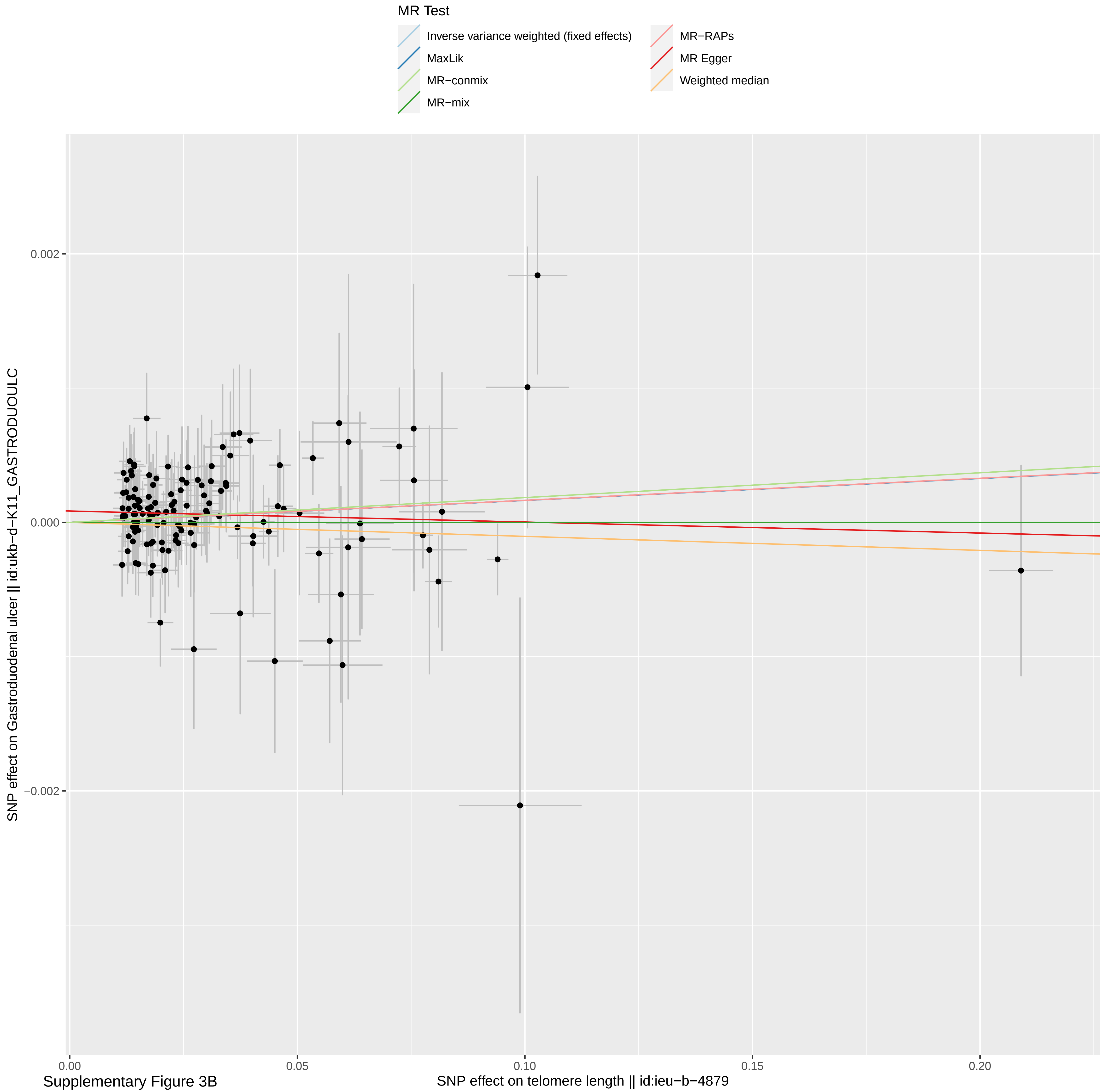

Supplementary Figure 3B

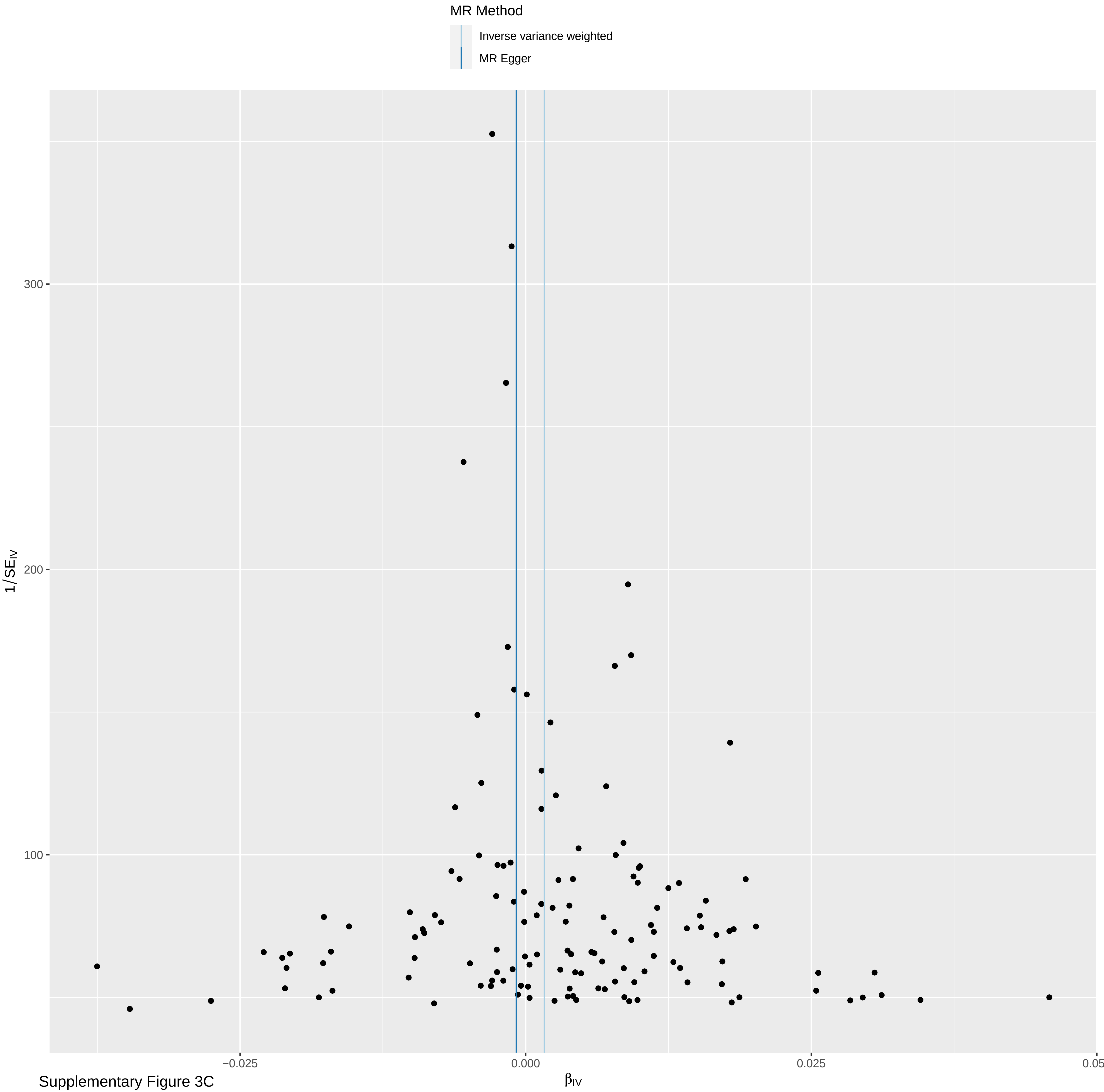

Supplementary Figure 3C

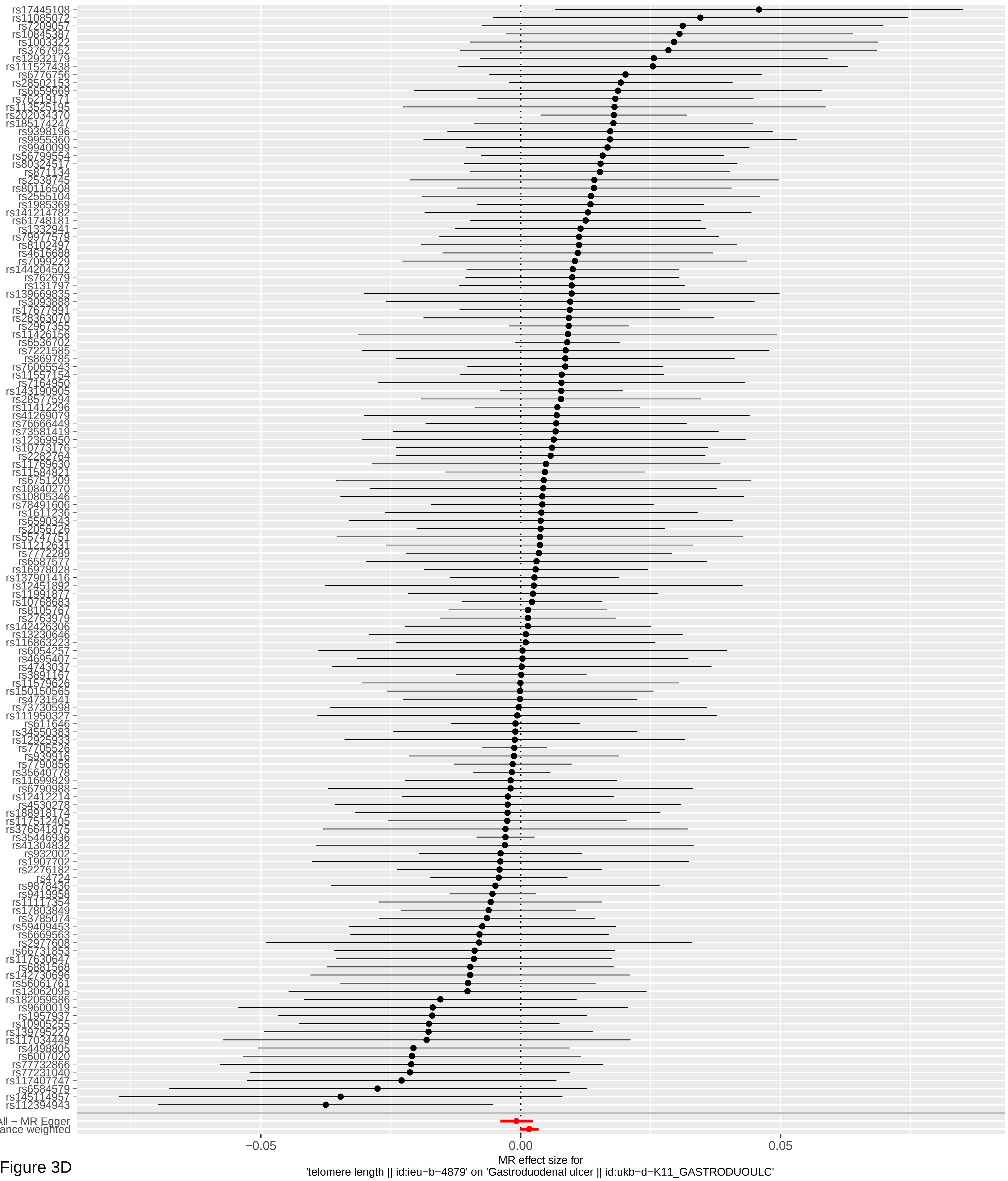

Supplementary Figure 3D

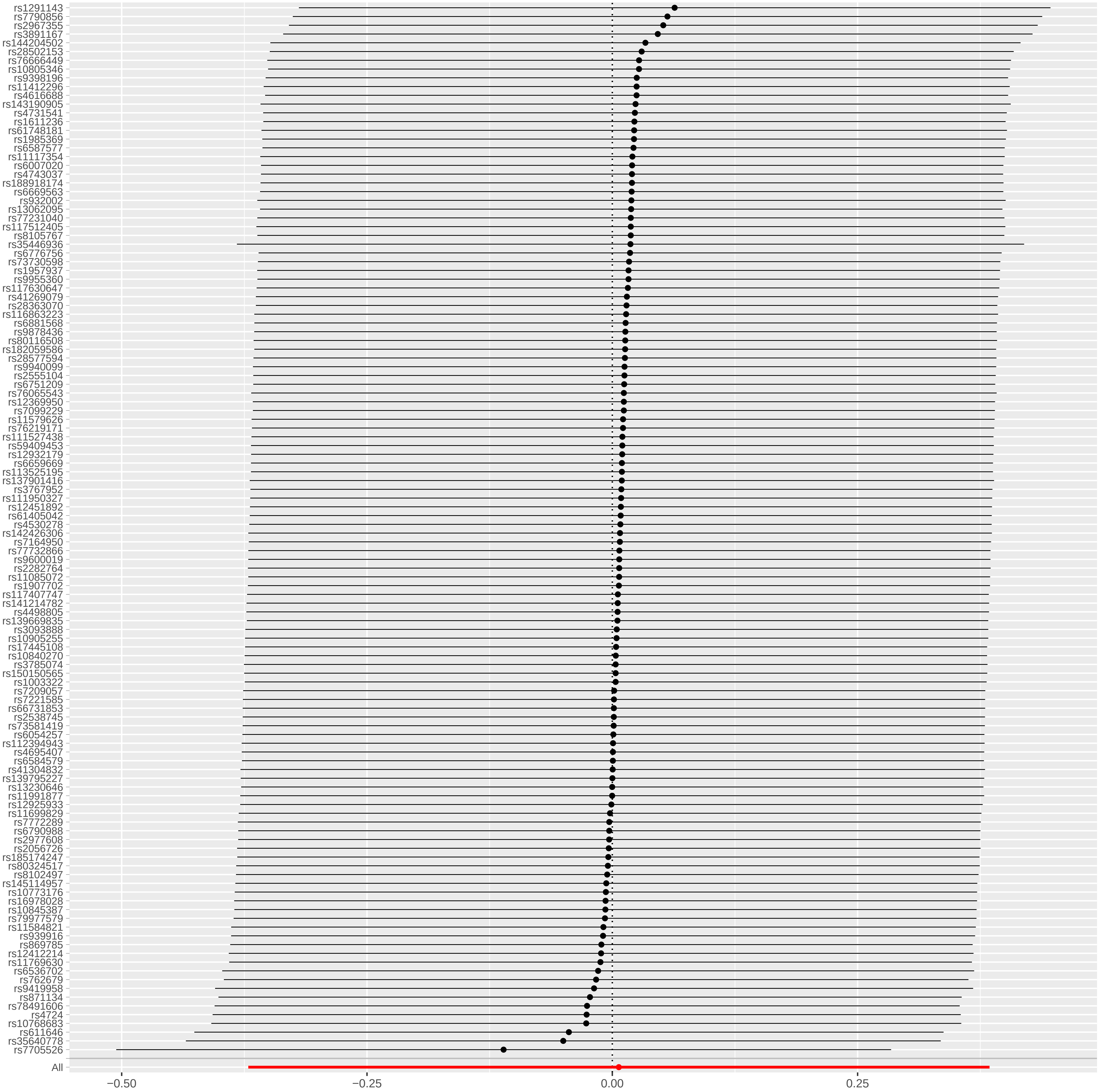

Supplementary Figure 4A

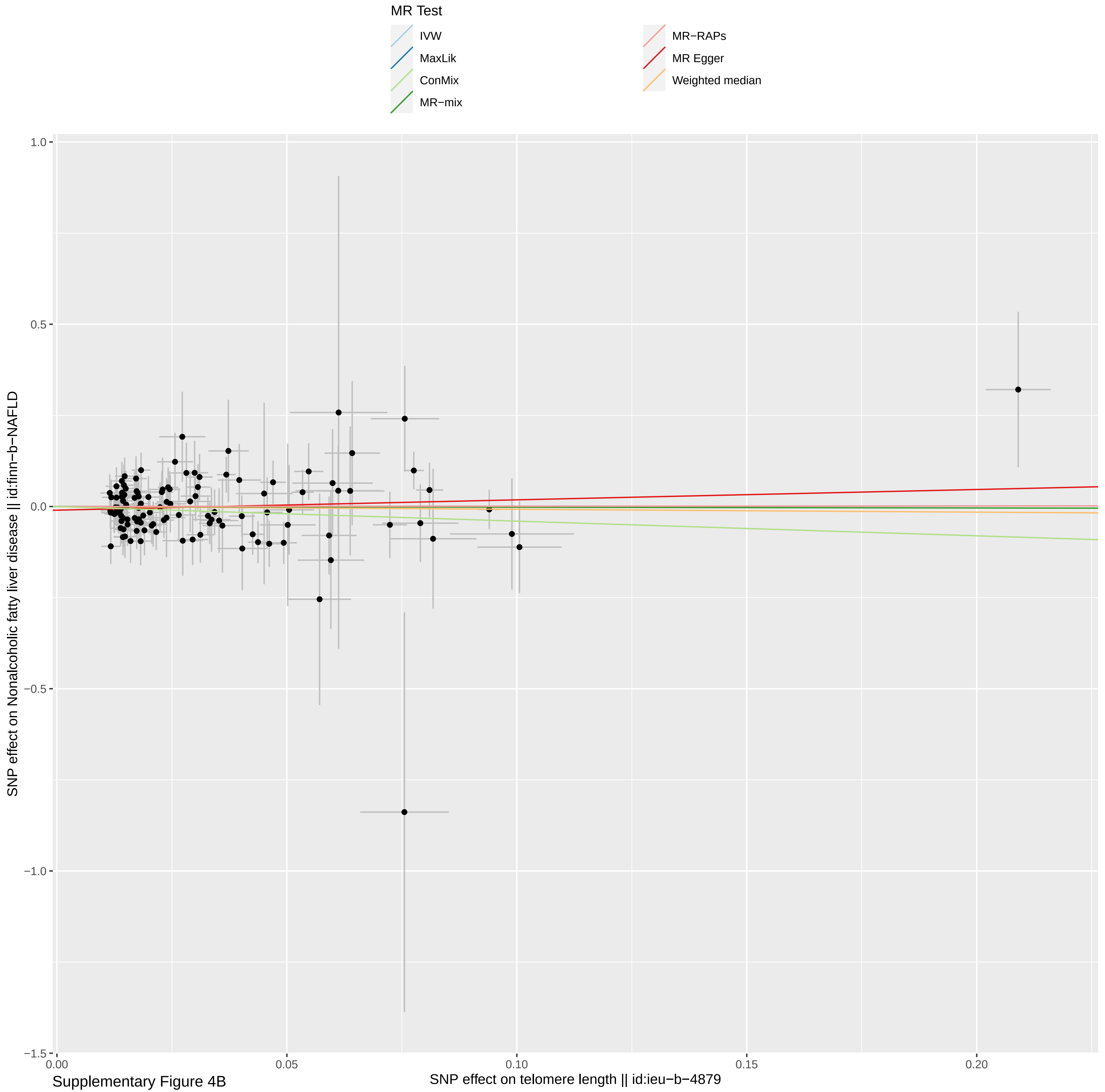

Supplementary Figure 4B

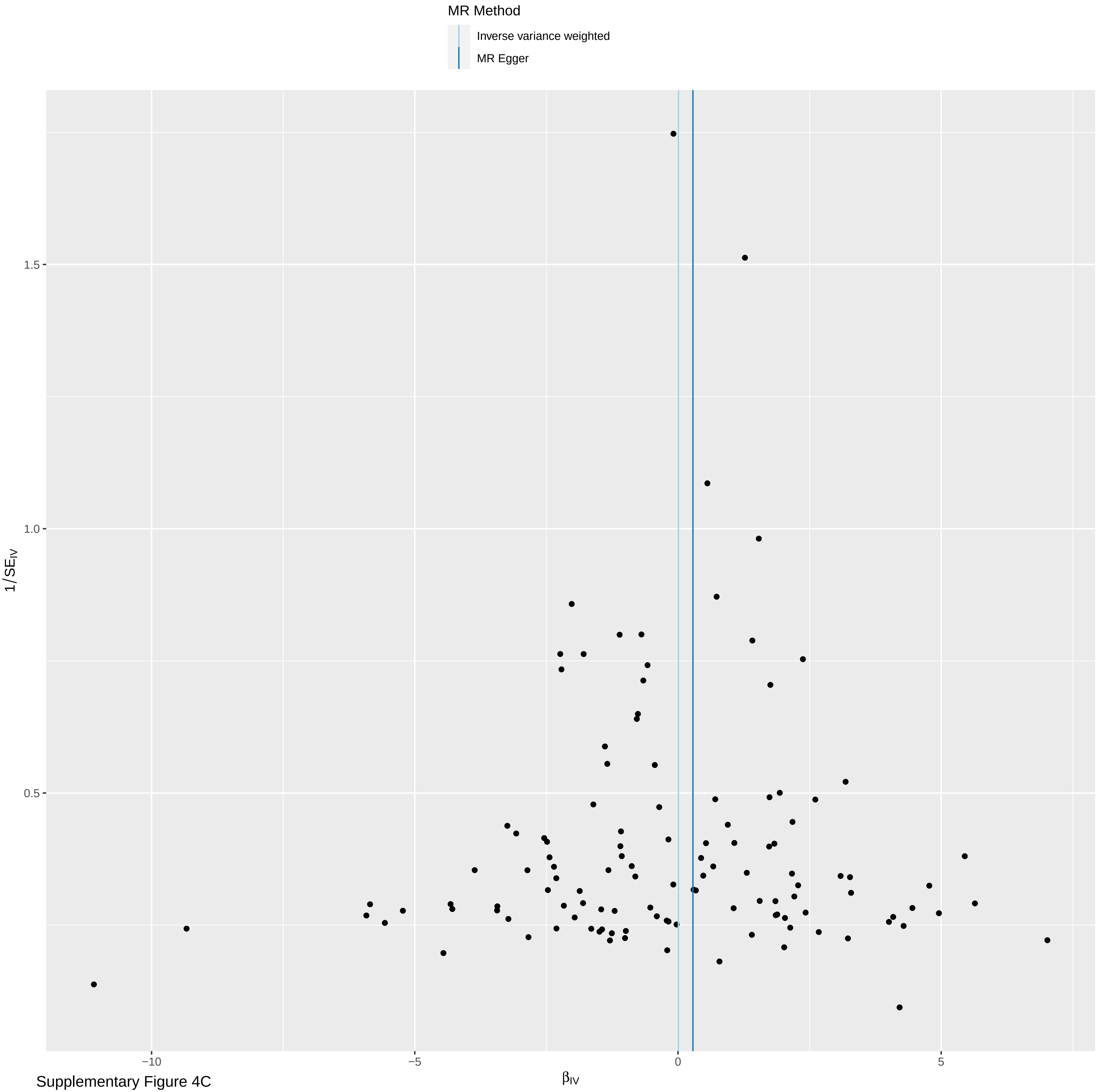

Supplementary Figure 4C

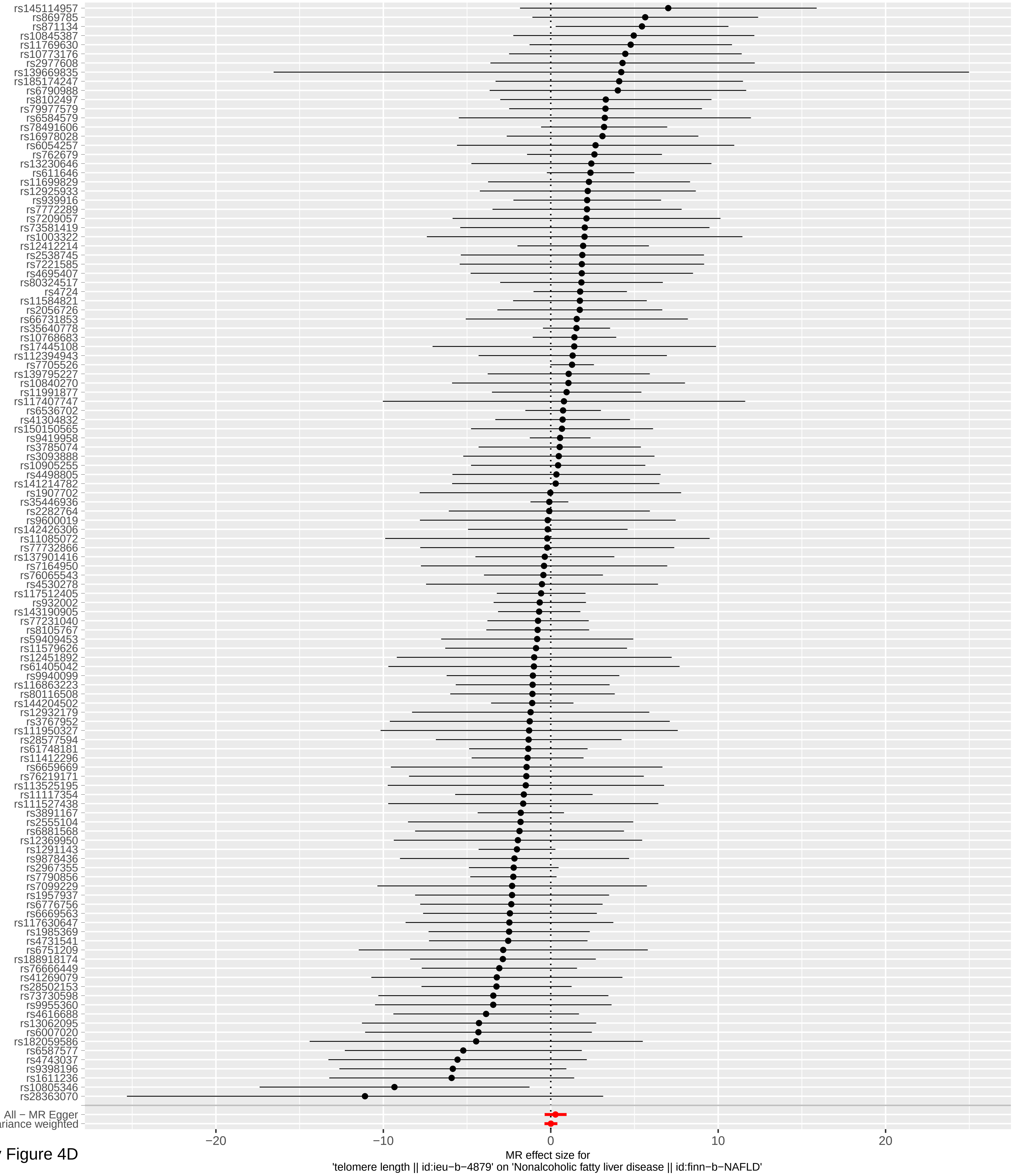

Supplementary Figure 4D

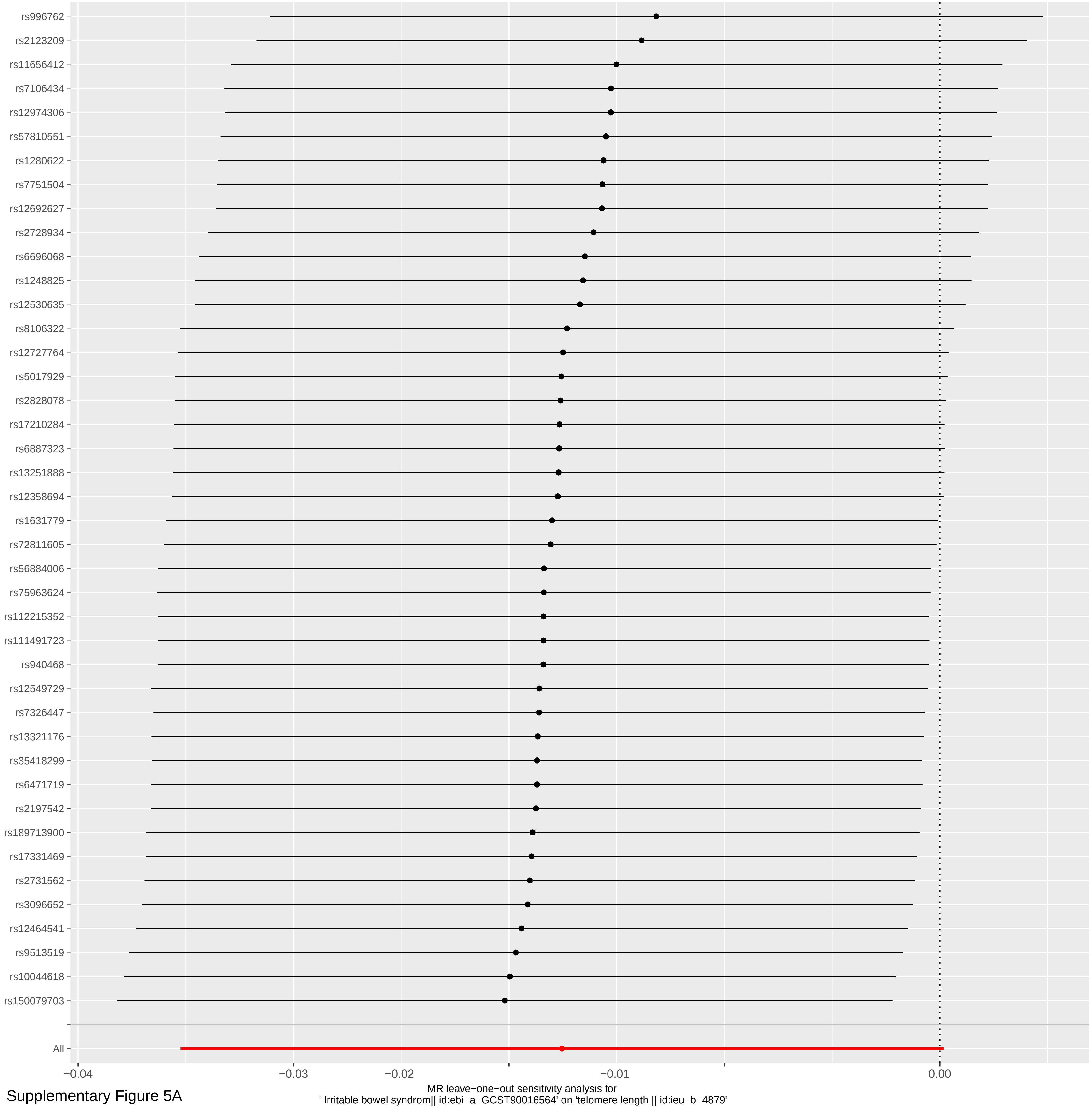

Supplementary Figure 5A

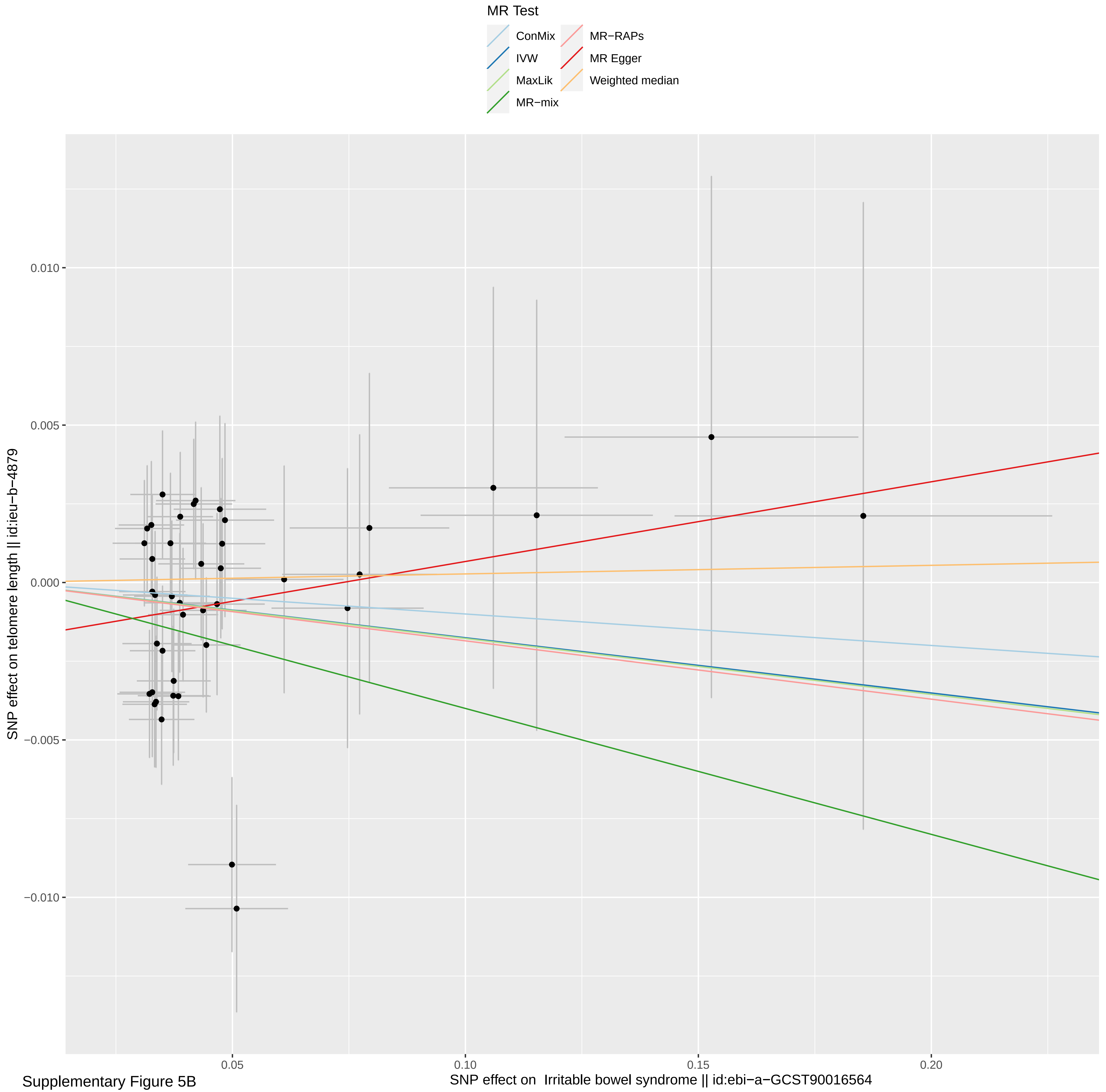

Supplementary Figure 5B

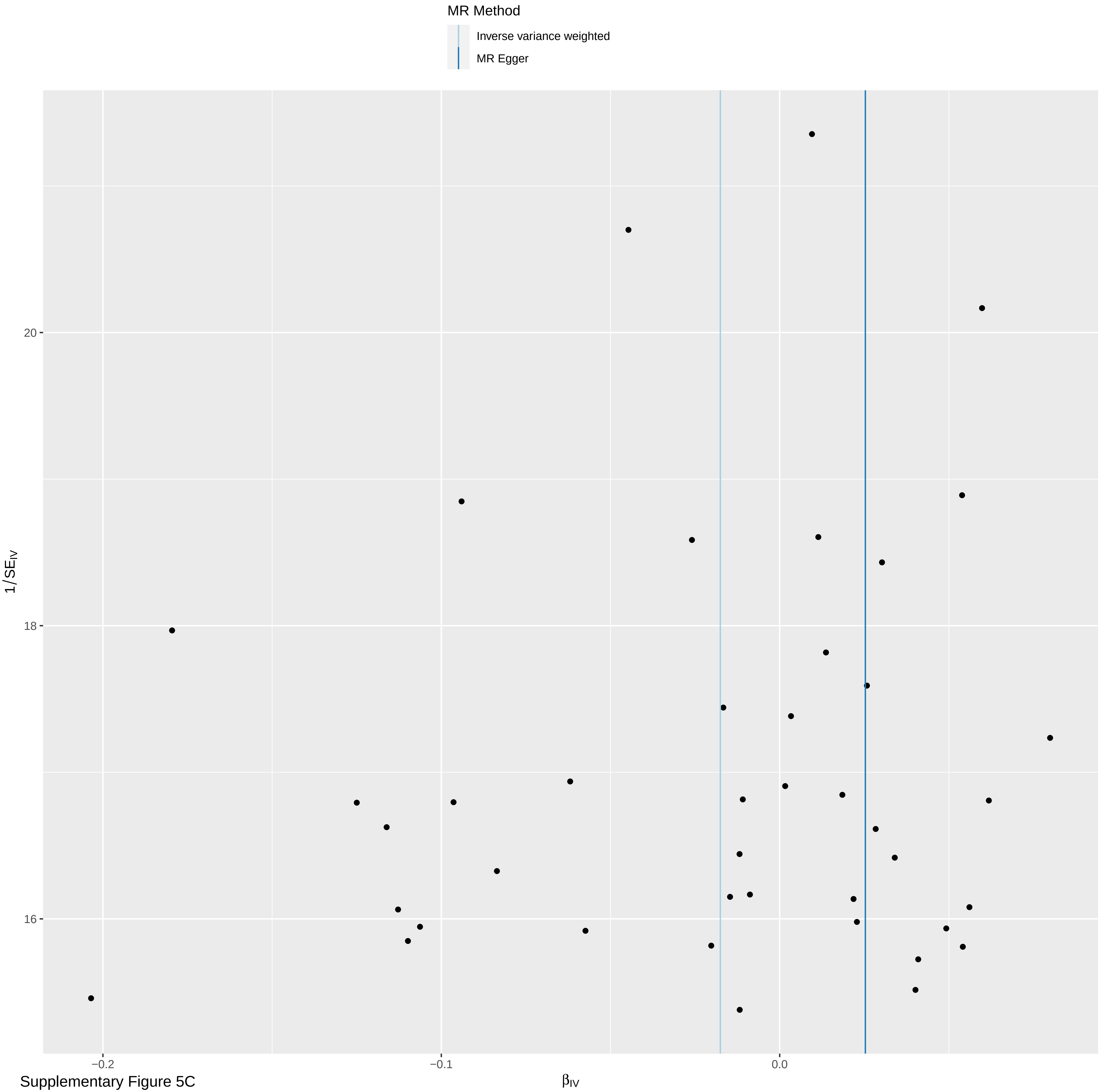

Supplementary Figure 5C

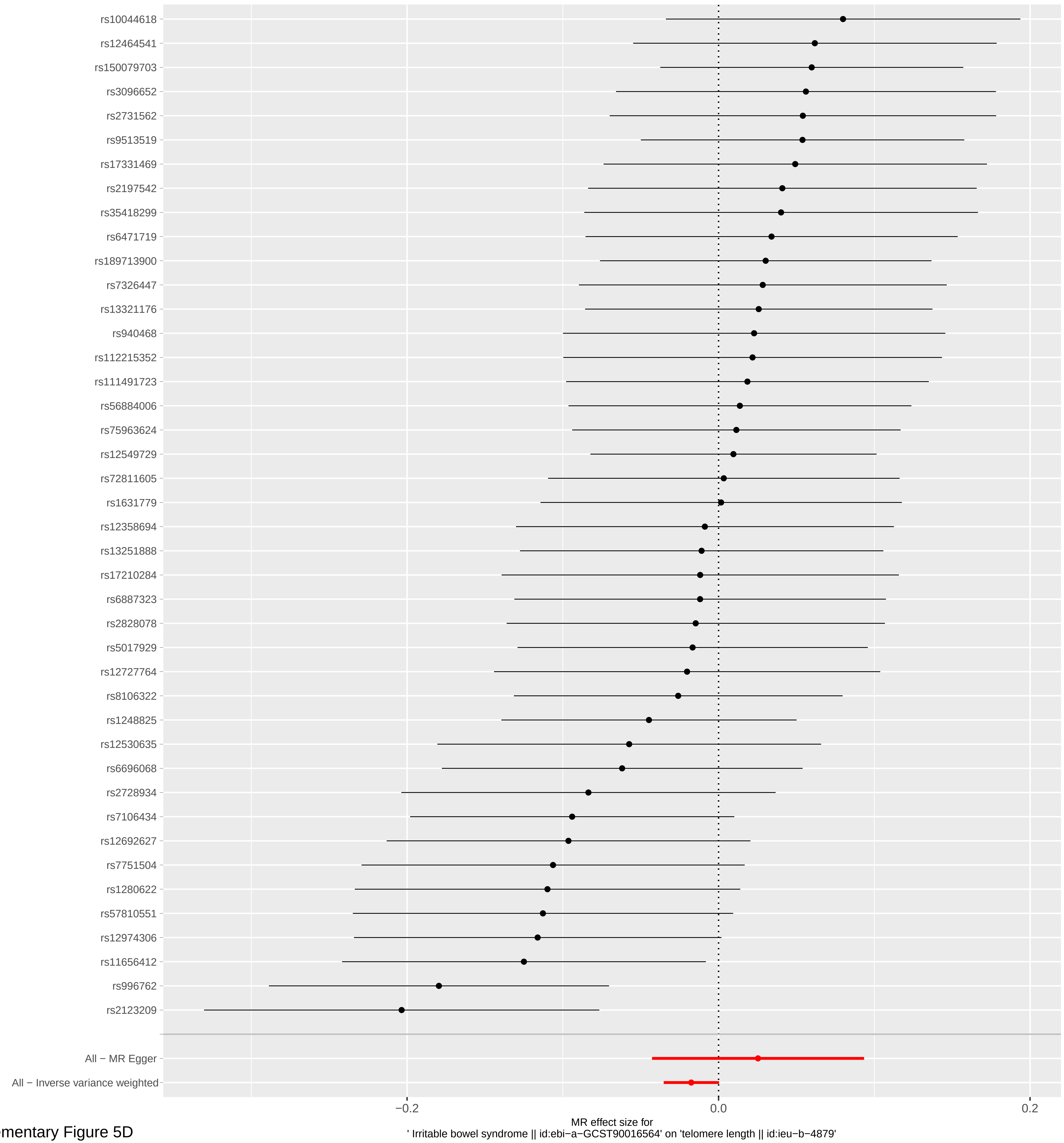

Supplementary Figure 5D

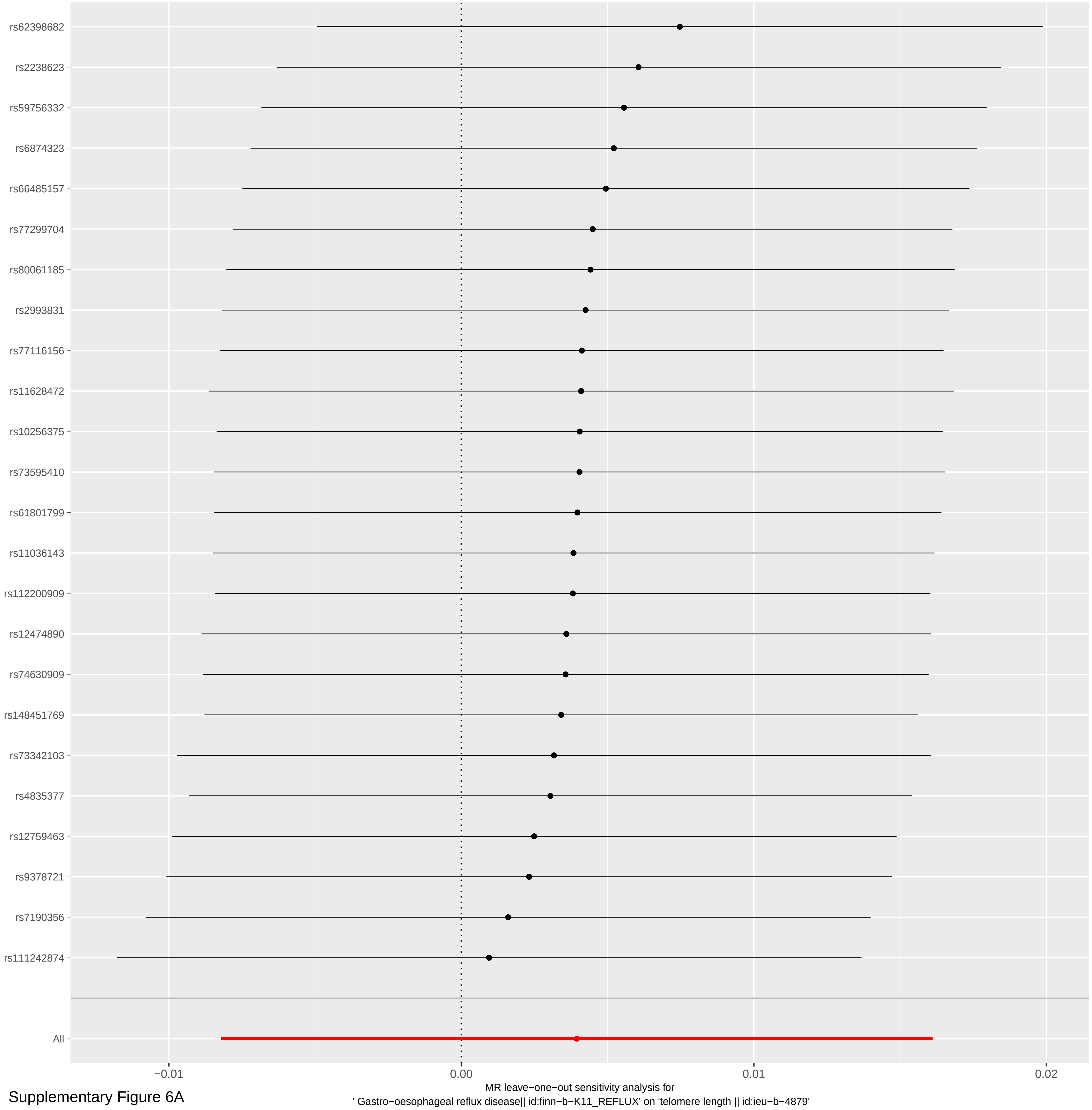

Supplementary Figure 6A

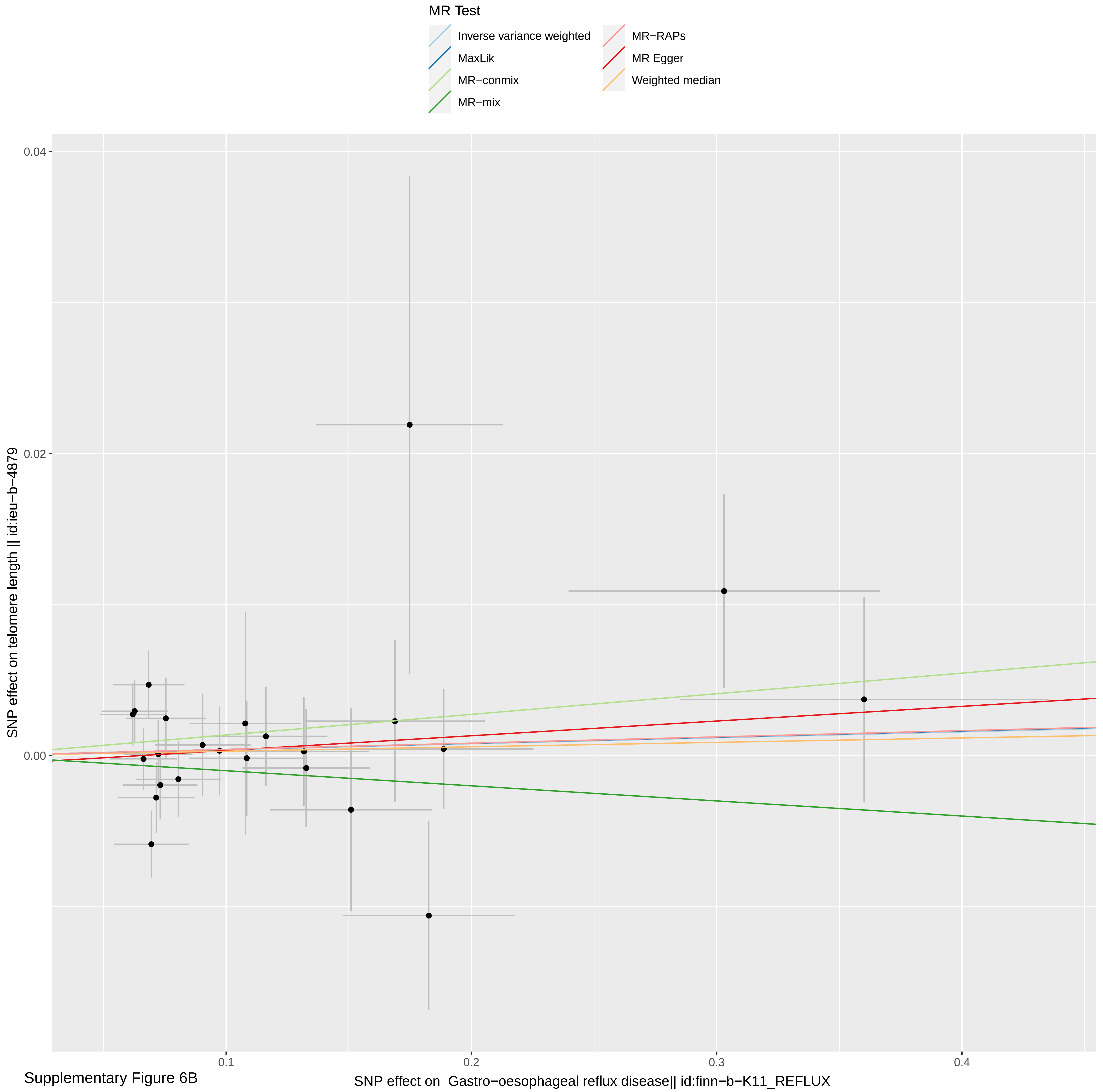

Supplementary Figure 6B

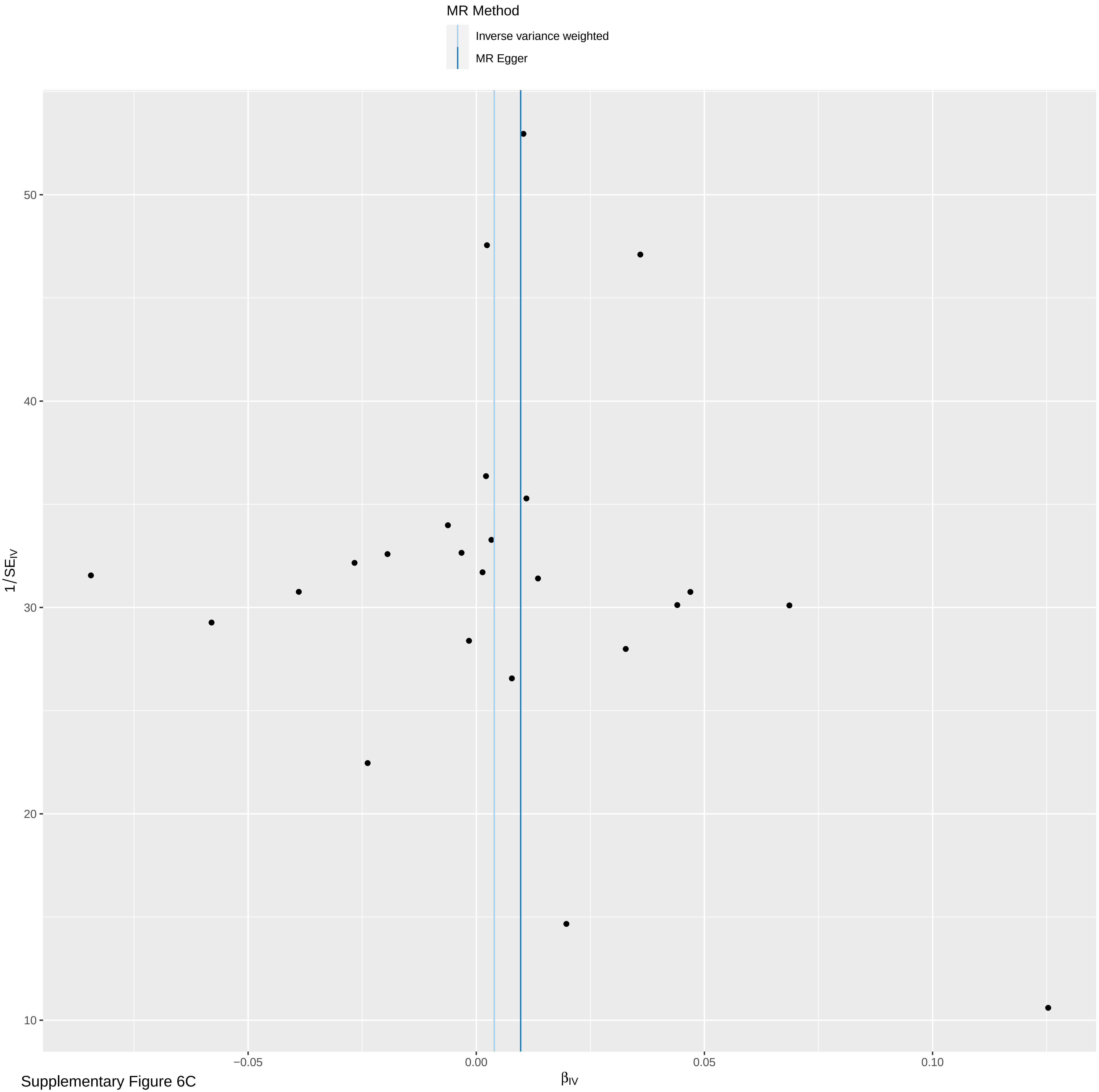

Supplementary Figure 6C

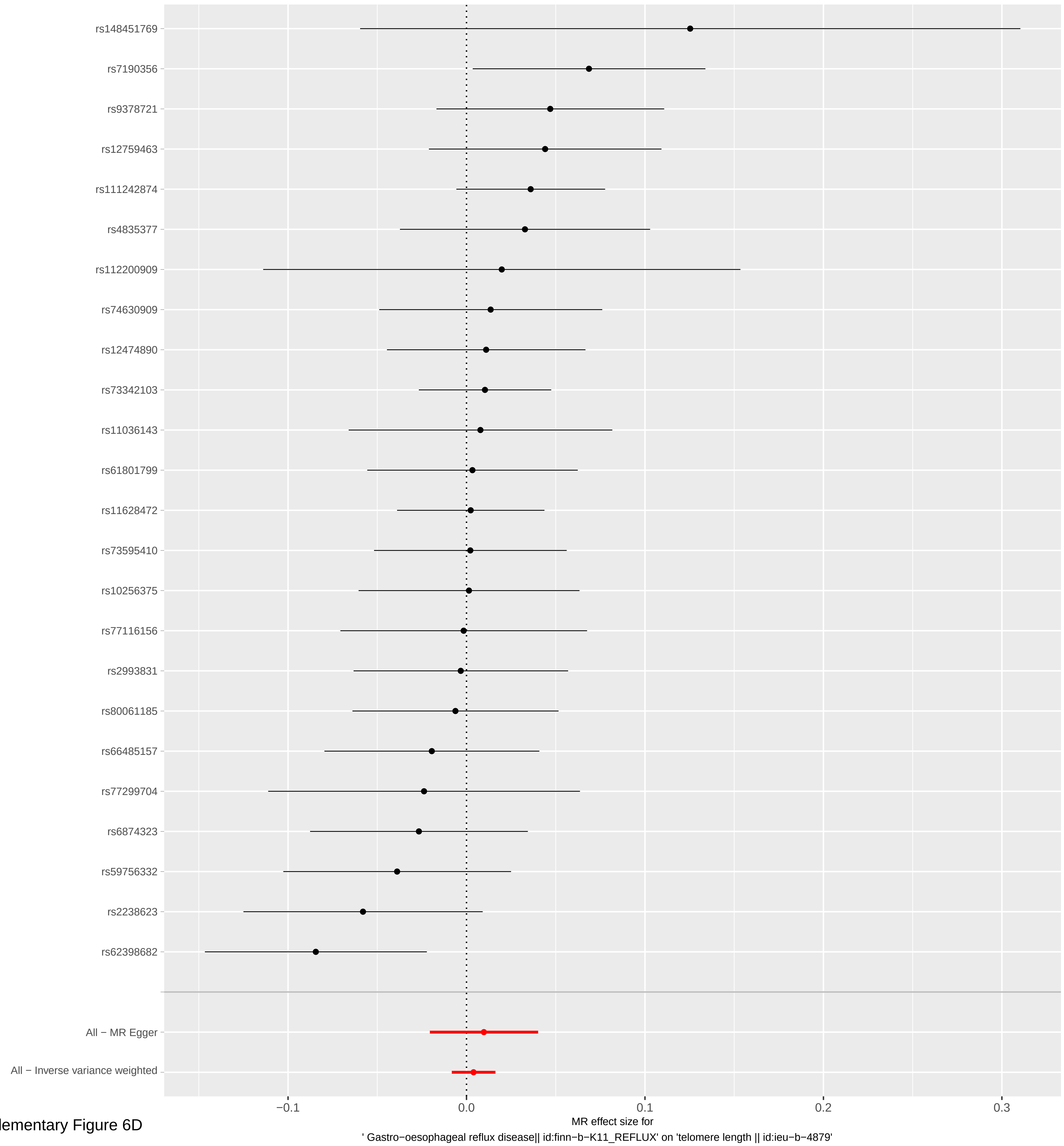

Supplementary Figure 6D

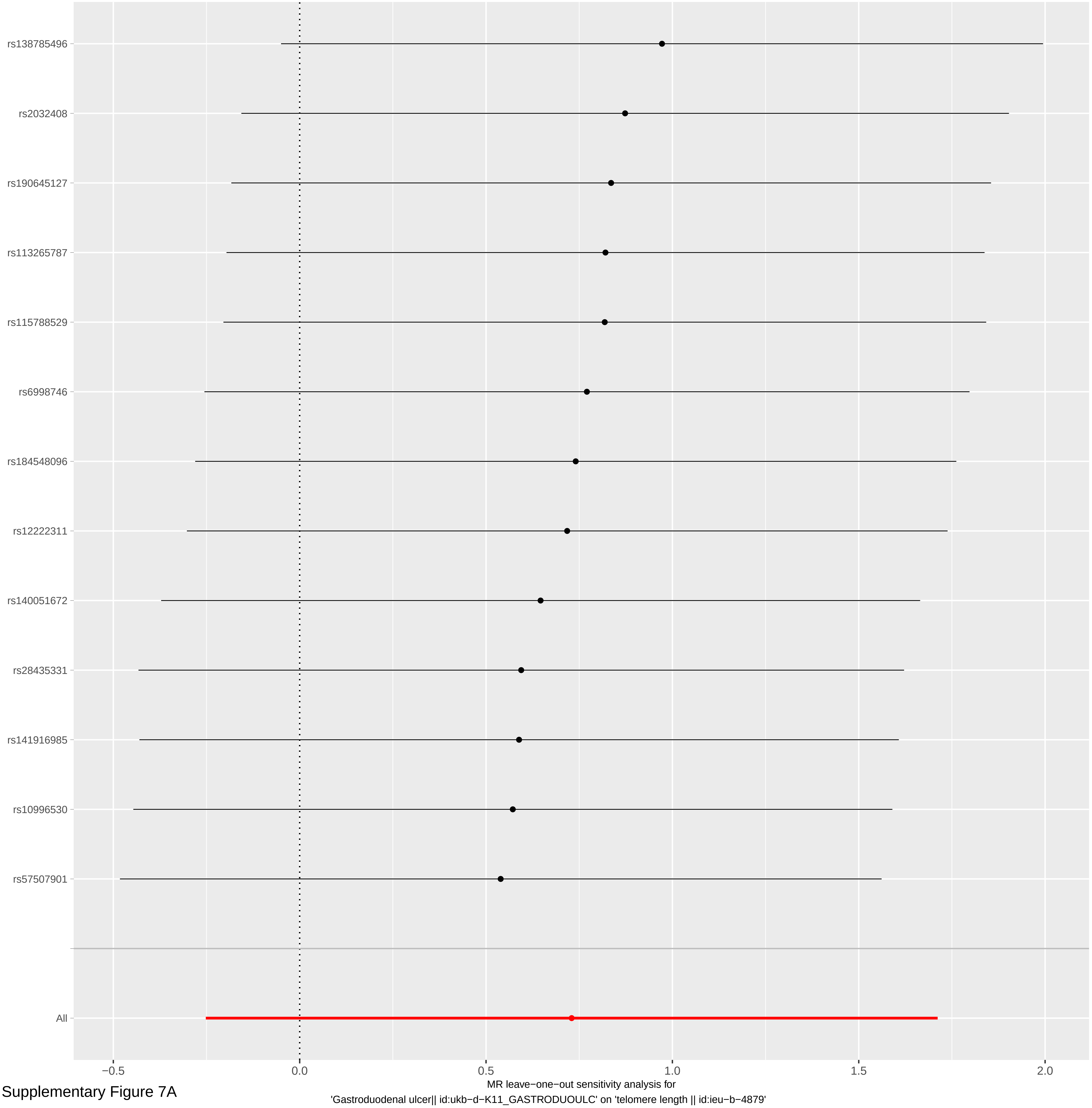

Supplementary Figure 7A

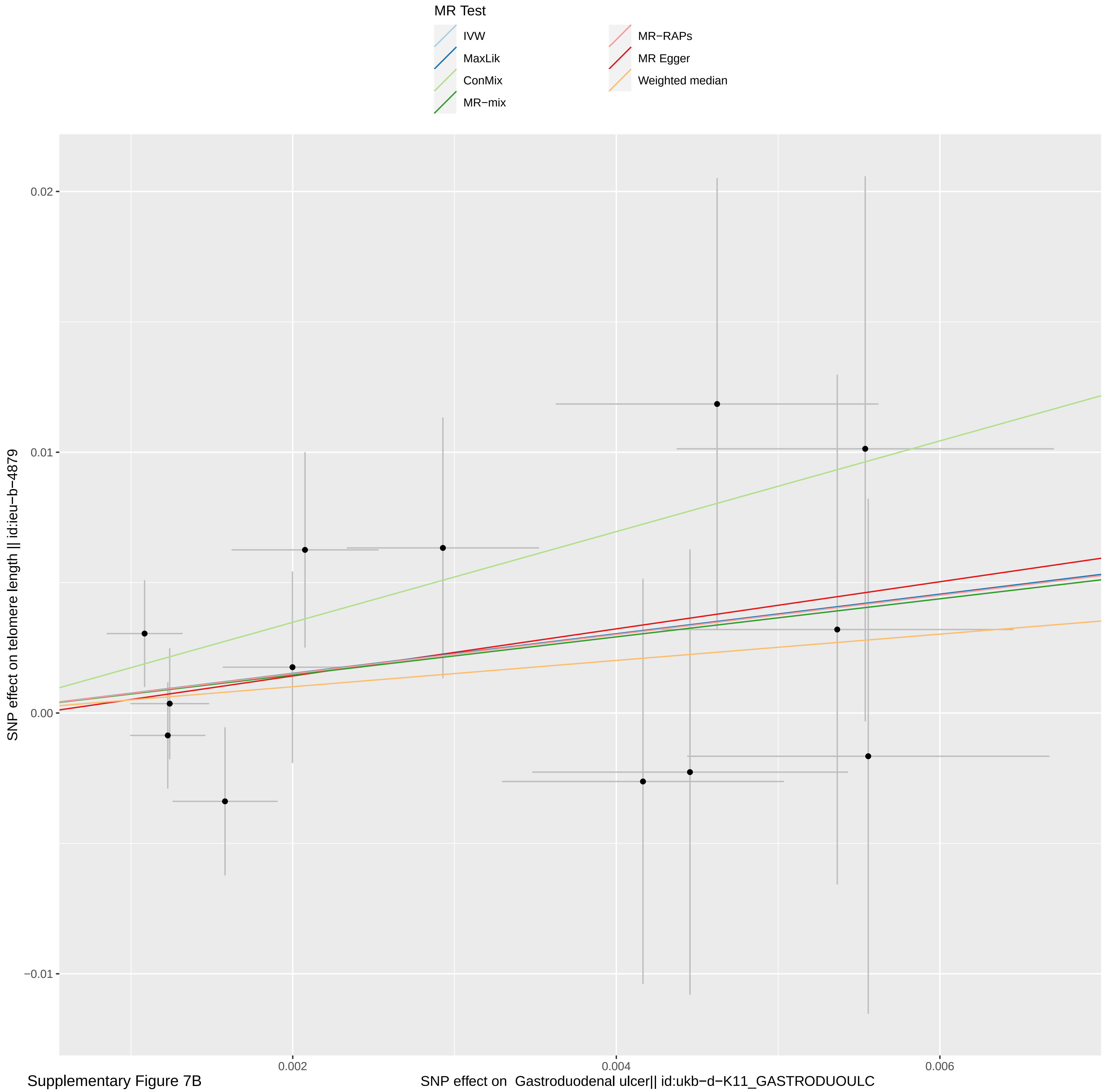

Supplementary Figure 7B

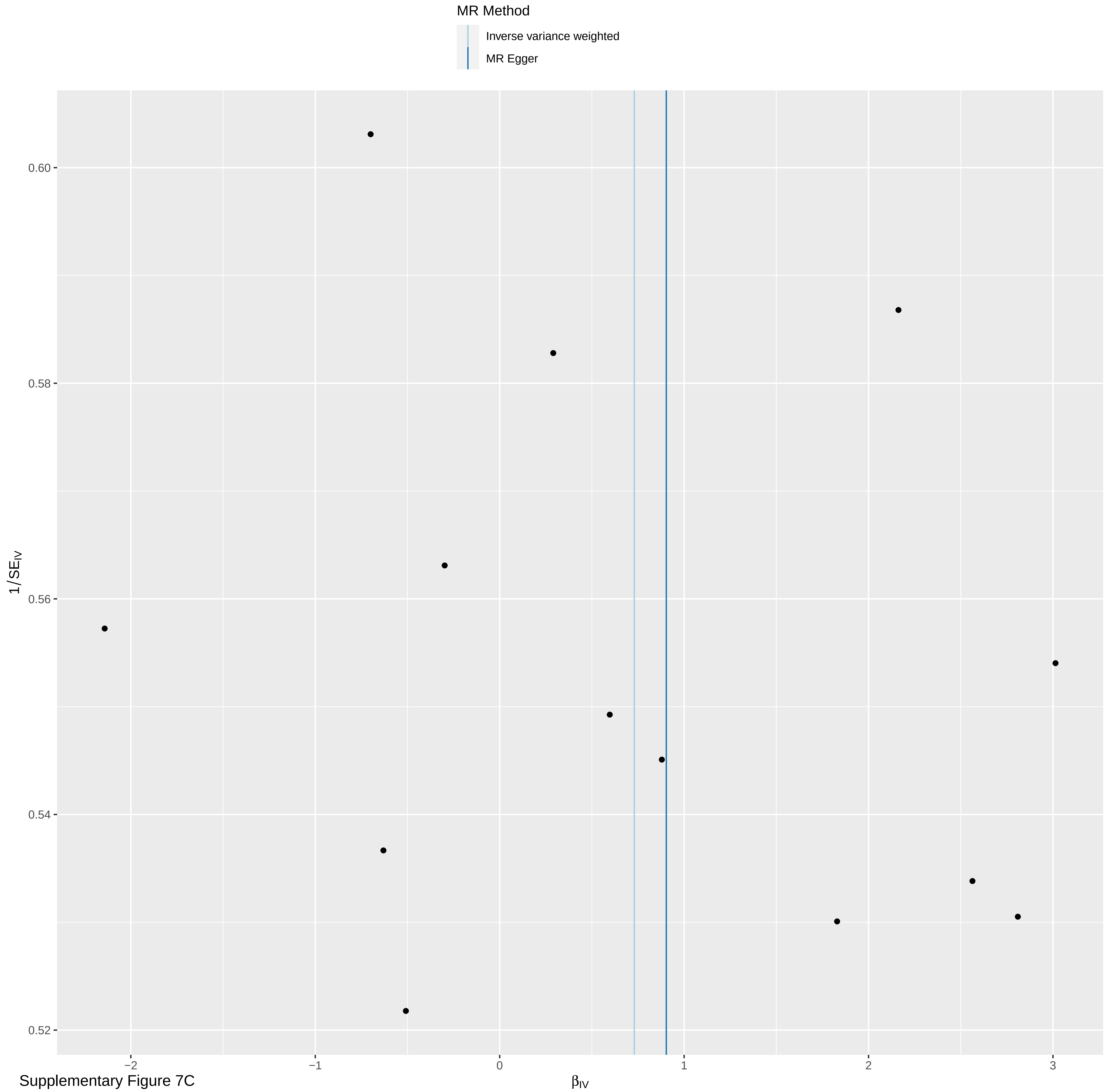

Supplementary Figure 7C

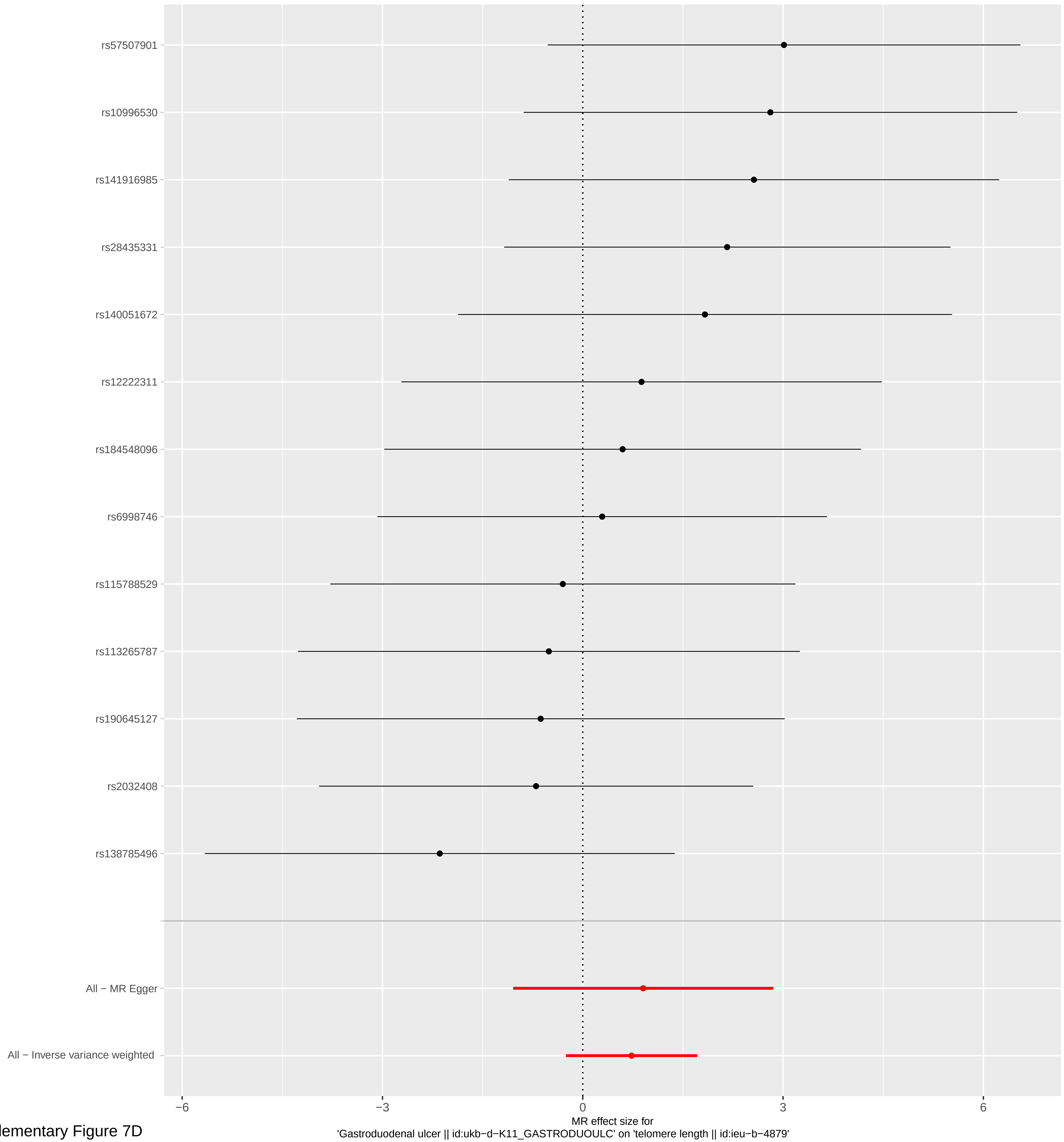

Supplementary Figure 7D

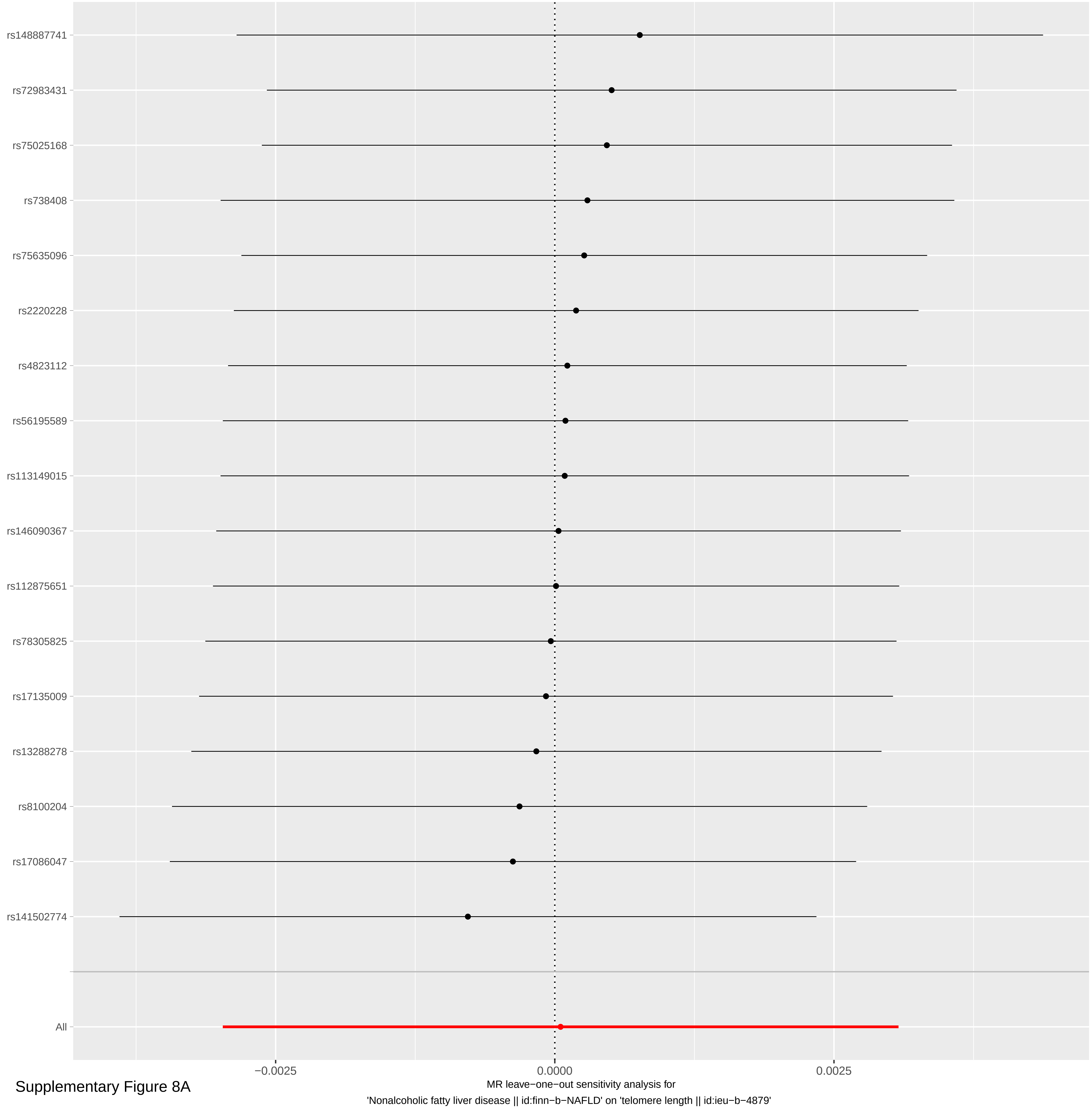

Supplementary Figure 8A

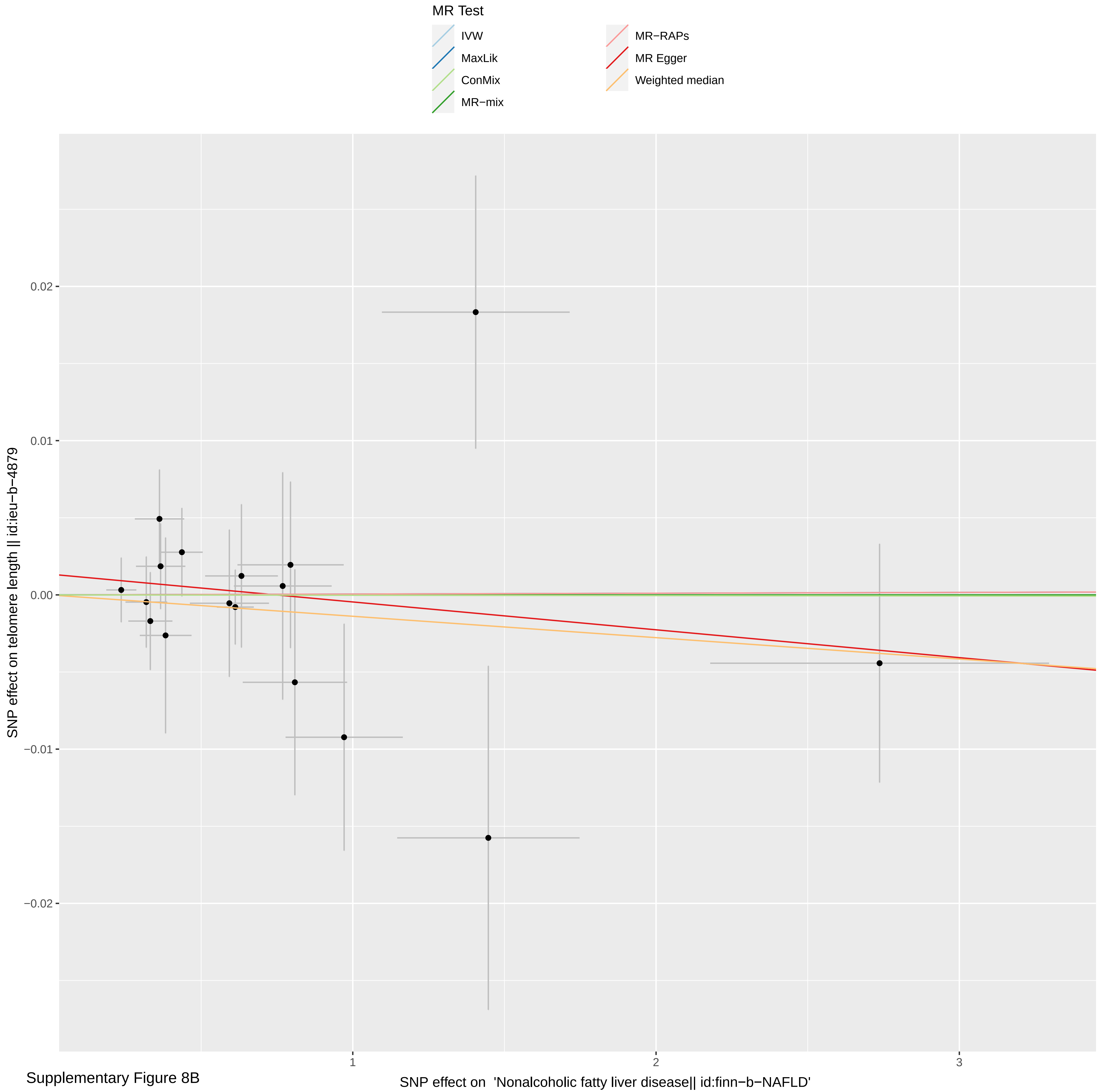

Supplementary Figure 8B

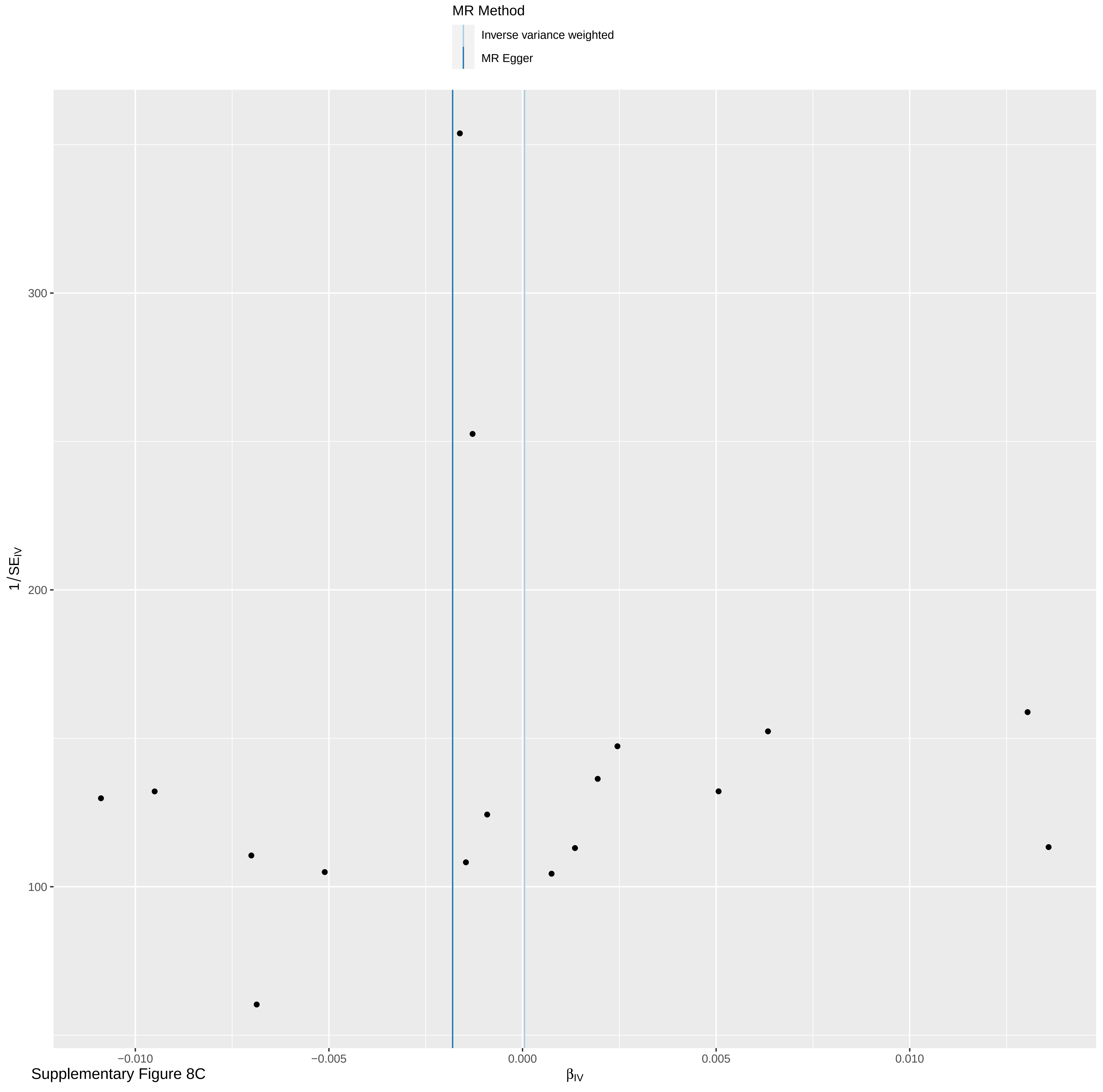

Supplementary Figure 8C

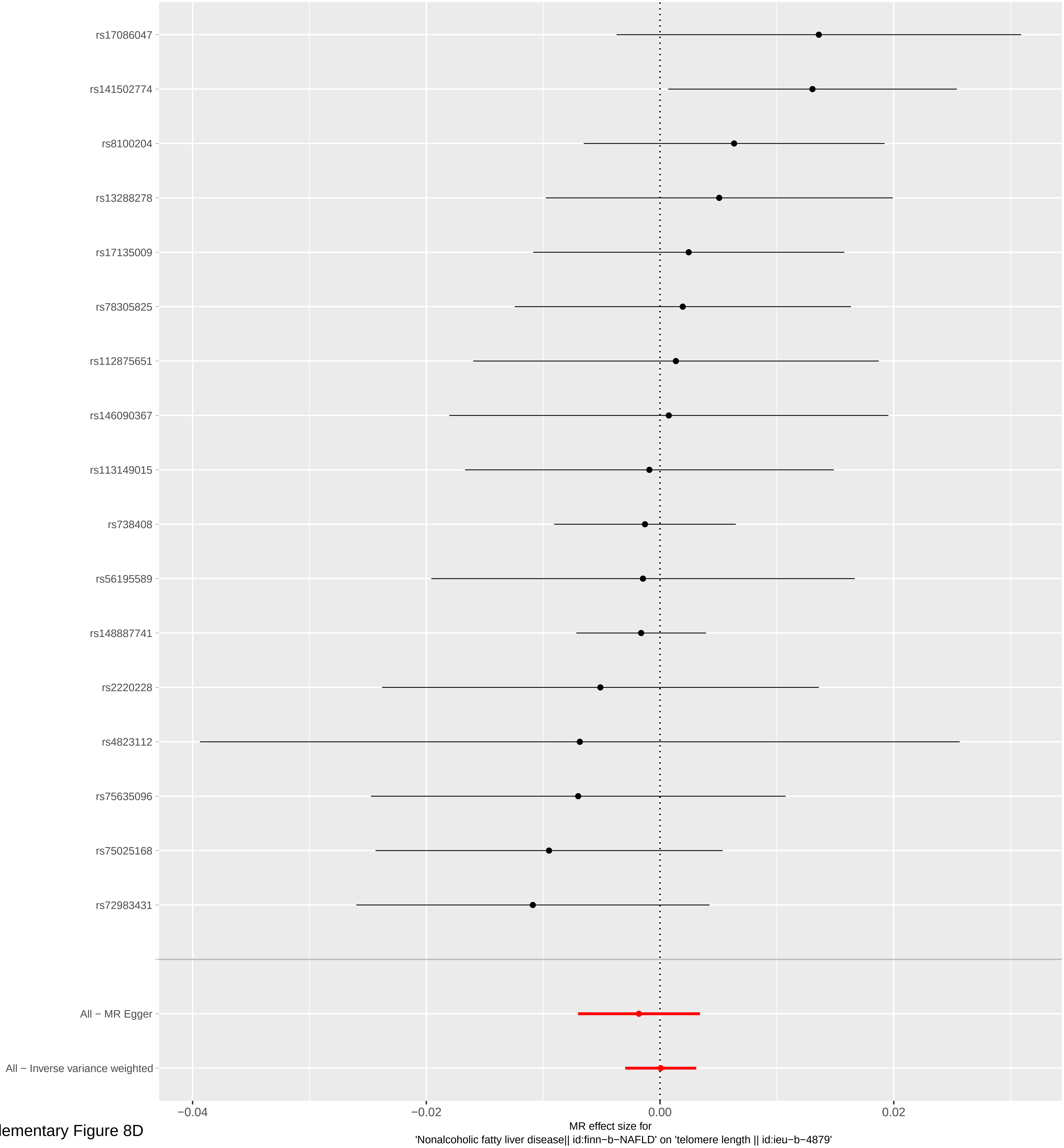

Supplementary Figure 8D
